# *Aconitum tanguticum* (Maxim.) Stapf.: a review of its traditional uses, chemical constituents, and pharmacological effects

**DOI:** 10.3389/fphar.2026.1811231

**Published:** 2026-08-21

**Authors:** Jian Wang, Xiujie Jin, Jinglong Wang, Jian Yu

**Affiliations:** 1 College of Food Science and Pharmaceutical Engineering, Zaozhuang University, Zaozhuang, China; 2 College of Pharmacy, Shandong University of Traditional Chinese Medicine, Jinan, China

**Keywords:** *Aconitum tanguticum* (Maxim.) Stapf., Bangga, chemical composition, ethnopharmacology, pharmacological action, Tibetan medicine, traditional use

## Abstract

*Aconitum tanguticum* (Maxim.) Stapf., commonly known as “Bangga” when used in combination with *Aconitum naviculare* (Brühl.) Stapf., is a traditional Tibetan medicine widely used in medicinal preparations. It is documented in the *Si-Bu-Yi-Dian* and *Jing-Zhu-Ben-Cao* and is mainly used to treat infectious and inflammatory conditions such as fever, liver and biliary disorders, lung diseases, gastroenteritis, influenza, and food poisoning. The primary bioactive ingredients of Bangga include diterpenoids, flavonoids, and phenolic acids. Additionally, extracts of Bangga have numerous beneficial biological activities, including antibacterial, anti-inflammatory, antiviral, and antitumor properties. The integration of traditional Tibetan medicinal formulas with modern pharmacological research can reveal new directions for clinical applications. For example, other formulas such as Ba-Wei-Zhang-Ya-Wan, An-Er-Ning-Ke-Li, and Er-Shi-Wu-Wei-Gui-Jiu-Wan have shown efficacy in treating cholecystitis, pediatric hand-foot-mouth disease and asthma, and osteoporosis, respectively. Previous phytochemical research has mainly focused on its alkaloids, flavonoids, phenolic acids, and volatile oils. Accordingly, this review presents a comprehensive overview of studies on the traditional uses, chemical composition, and modern pharmacological effects of *Aconitum tanguticum* (Maxim.) Stapf. This review systematically summarizes the multi-target pharmacological mechanisms of its alkaloids, flavonoids, and phenolic acids, highlights its therapeutic potential in treating inflammatory bowel disease, cardiovascular diseases, and acute lung injury, and provides a theoretical basis for the integration of this ethnic medicine into modern healthcare. Furthermore, it emphasizes the need for further research to facilitate its translation into clinical practice and advance its global recognition as a promising therapeutic agent.

## Graphical Abstract

**Figure:**
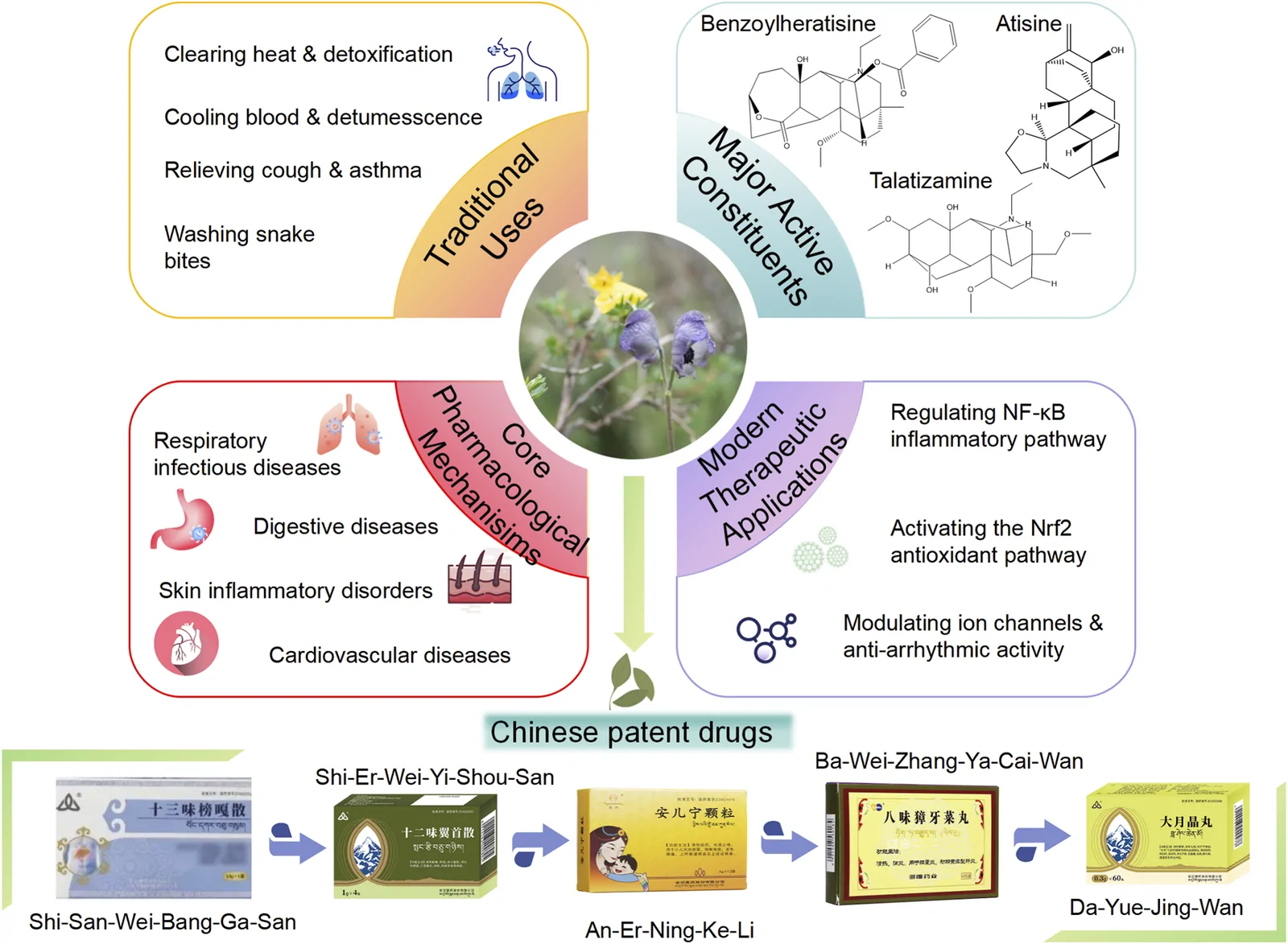

## 1 Introduction

*Aconitum tanguticum* (Maxim.) Stapf. (ATS), also known by its Tibetan name “Bangga” when combined with *Aconitum naviculare* (Brühl.) Stapf., is commonly used in traditional medicinal preparations (National Pharmacopoeia Commission, 2020). The plant grows at an altitude of 3,200–4,800 m in alpine meadows and gravel belts and is distributed in Sichuan, Yunnan, Tibet, Qinling, Gansu, and Qinghai. The dried plants are conventionally used for treating liver fever, bile fever, lung fever, hepatitis, pneumonia, gastroenteritis, influenza, and similar infectious conditions.

**FIGURE 1:**
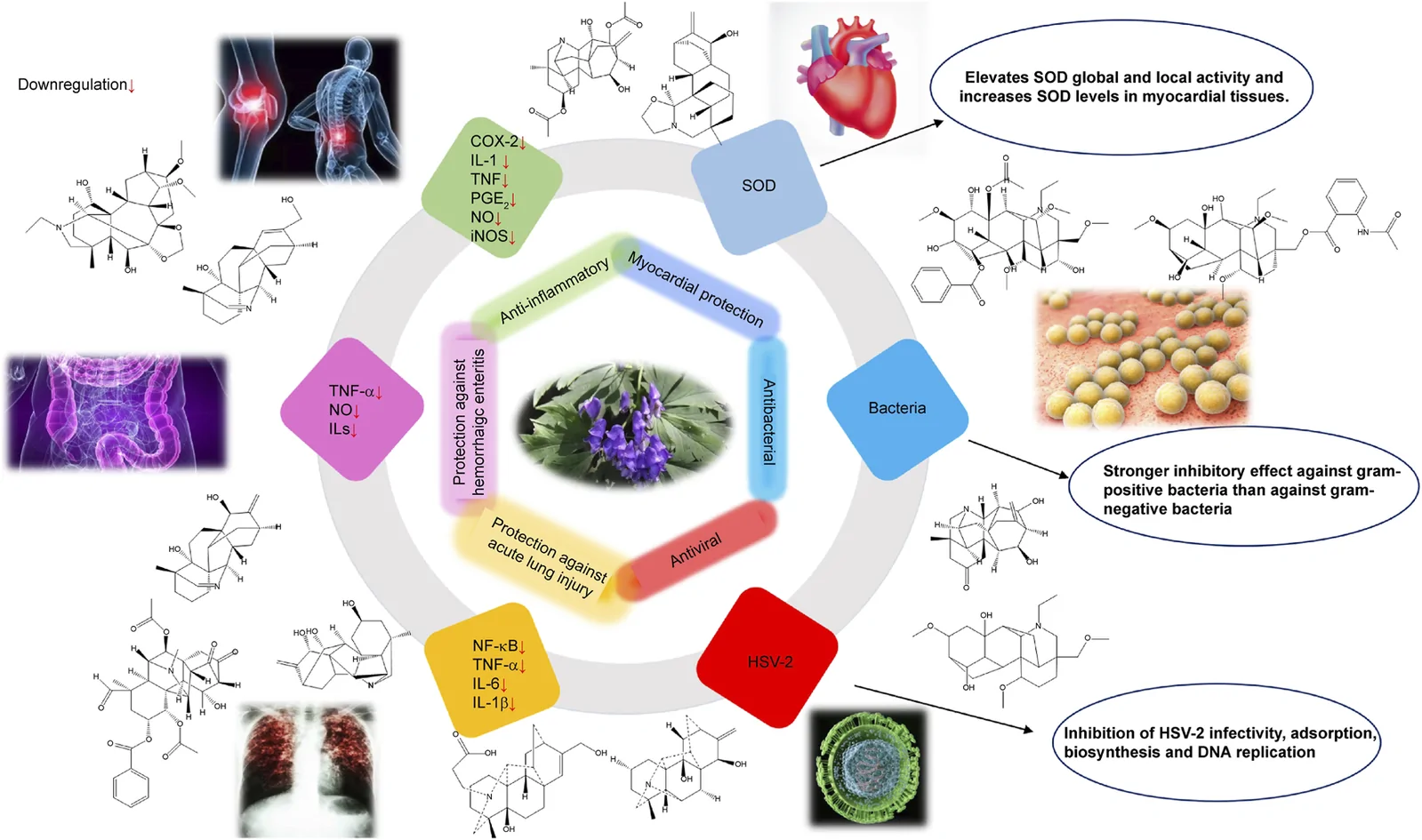
Pharmacological activities of *Aconitum tanguticum* (Maxim.) Stapf. Abbreviations: COX-2, Cyclooxygenase-2; IL-1, Interleukin-1; TNF, Tumor necrosis factor; PGE_2_, Prostaglandin E_2_; iNOS, Inducible nitric oxide synthase; TNF-α, Tumor necrosis factor-alpha; NO, Nitric oxide; ILs, Interleukins; NF-κB, Nuclear factor-kappa B; IL-6, Interleukin-6; IL-1β, Interleukin-1 beta; SOD, Superoxide dismutase; HSV-2, Herpes simplex virus type 2.

ATS, a Tibetan medicine, induces anti-inflammatory, analgesic, sedative, antipyretic, immunosuppressive, antitumor, cardiac-strengthening, blood pressure-lowering, and blood vessel-dilating effects (Figure 1). However, systematic studies on ATS remain fragmented, thereby yielding a limited understanding of the association between its traditional therapeutic applications and modern pharmacological mechanisms. To the best of our knowledge, no comprehensive reviews have systematically evaluated the clinical efficacy, phytochemical diversity, or pharmacological effects of ATS.

In this review, we systematically synthesized the traditional uses, chemical constituents (such as alkaloids, flavonoids, and phenolic acids), and pharmacological effects of ATS to clarify its core value in traditional prescriptions and reveal its potential clinical mechanisms. Therefore, this study emphasizes the necessity of medicinally exploring ATS to aid in its transition from a local traditional medicine to an internationally recognized innovative drug, thereby providing theoretical support for its modernization.

## 2 Methods

A literature review methodology was employed using search terms such as “*Aconitum tanguticum* (Maxim.) Stapf.,” “Bangga,” “chemical constituents,” “pharmacological effects,” and “Tibetan medicine” to conduct a comprehensive search of multiple literature databases, including CNKI, PubMed, Wanfang Data, and Baidu Scholar. The search period covers the time from the inception of each database to the completion of the literature review. Chinese and English full texts related to the plant’s chemical composition, pharmacological activity, and traditional application were included, while duplicate records, abstracts, patents, and irrelevant documents were excluded. Literature was screened by title, abstract, and full text. The quality of pharmacological studies was assessed based on research design, sample size, and methodological integrity, and eligible literature was sorted and analyzed.

## 3 Botanical characteristics

**FIGURE 2:**
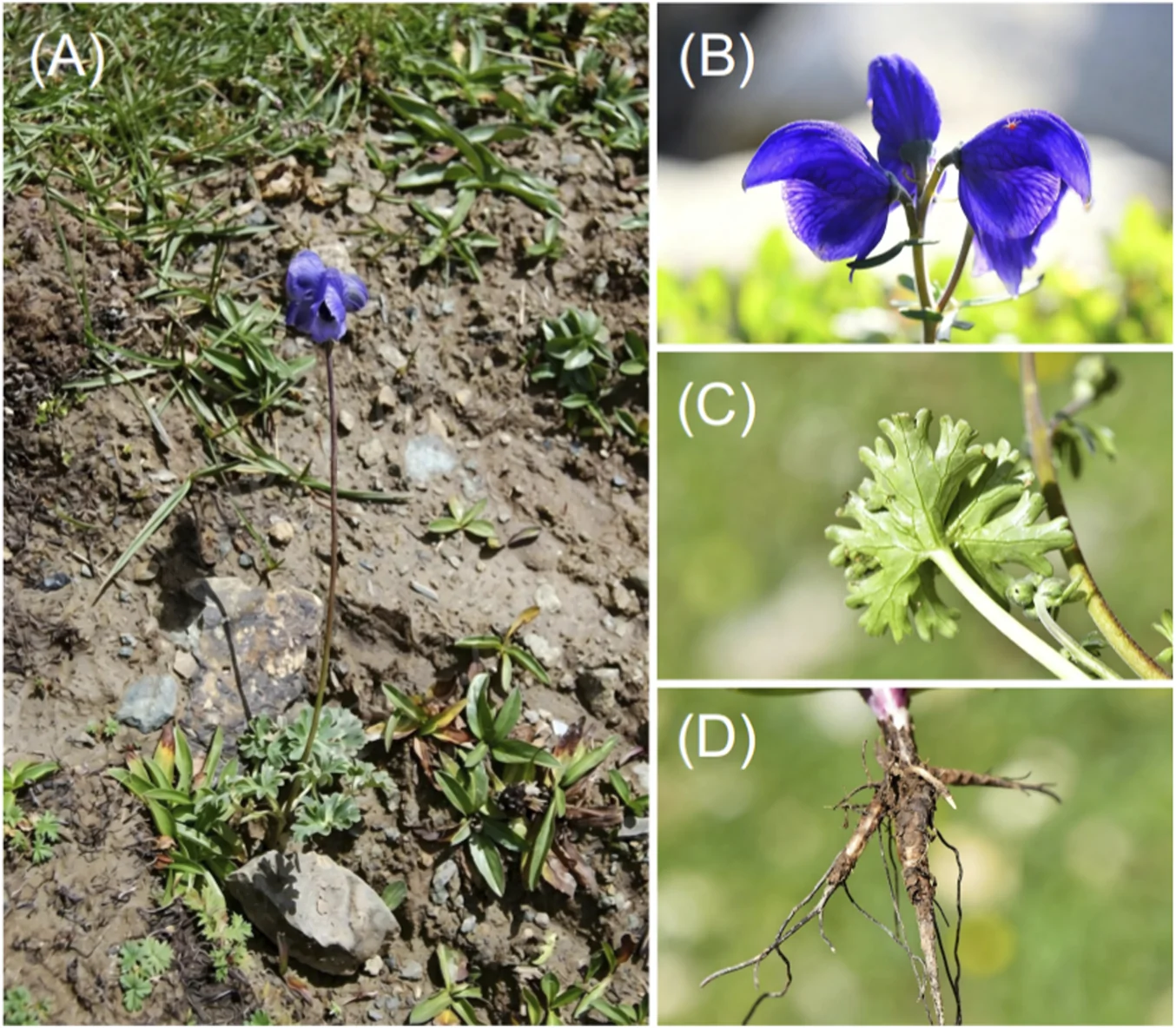
Botanical characteristics of *Aconitum tanguticum* (Maxim.) Stapf (ATS). **(A)** Whole plant; **(B)** Flowers; **(C)** Leaves; **(D)** Roots (Source: https://ppbc.iplant.cn).

ATS, from the buttercup family Ranunculaceae, is a perennial herbaceous plant used as a botanical drug. Its roots are small and spindle-shaped. Stems are 8–30 cm tall, sparsely puberulous or hairless (Wang, 2024), with up to nine basal leaves and long petioles. Leaf blades are contoured, rounded, or reniform, 1–3 cm long and 2–6.8 cm wide, with three deeply divided, central lobes that are fan-obovate, with lobed and rounded teeth. Cauline leaves are 1–4 in number and are relatively small. The inflorescence includes 3–5 flowers that are recurved and puberulent. Bracteoles are narrowly ovate to broadly striate. Sepals, which are present in groups of five, are blue-purple; the upper sepals are ship-shaped, with a lower margin 1.4–2.2 cm long. Petals are present in groups of two, glabrous, 0.6–1.5 mm long, and are either short or absent. There are multiple stamens and five glabrous carpels (Figure 2).

## 4 Traditional uses

ATS was first published in the book of ancient Tibetan medicine *Yue-Wang-Yao-Zhen* and was subsequently described in the *Si-Bu-Yi-Dian*, *Si-Bu-Yi-Dian-Zhu-Shi-Ben*, and *Jing-Zhu-Ben-Cao*. In 1996, the Qinghai Provincial Drug Inspection Institute and Tibetan Medicine Research Institute jointly issued the *Lan-Liu-Li*, which clearly describes its botanical characteristics: “The basic leaves of this product are three or four, the stem leaves are slightly small, and the deep split leaves are seven or eight; the stem is small and soft, and the flowers have a blue-red luster.” Moreover, it reports the core effect, namely, “detoxifying viruses and clearing bile heat.” The *Jing-Zhu-Ben-Cao* (Mao, 1986) further emphasizes its clinical value as follows: “detoxifying plague, clearing biliousness and fever, and detoxifying snake venom.” Modern research has confirmed that ATS is rich in alkaloid components and delivers anti-inflammatory and antibacterial effects, providing a scientific premise for its modernization.

**FIGURE 3:**
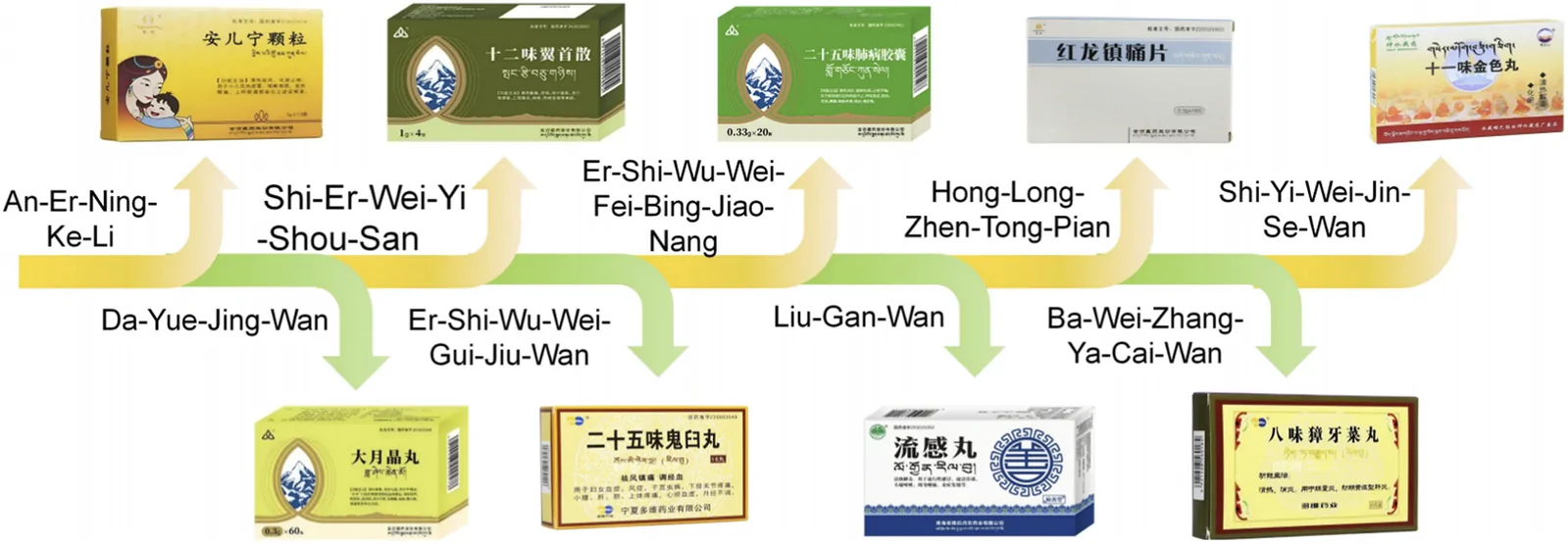
Schematic diagram of *Aconitum tanguticum* (Maxim.) Stapf. Compound preparation drug box.

Furthermore, ATS can be combined with other materials to achieve therapeutic effects. According to the *Yue-Wang-Yao-Zhen*, it can be used with *Punica granatum* L. (Lythraceae) and *Aconitum naviculare* (Brühl.) Stapf., *Aegle marmelos* (L.) Correa (Rutaceae), and gallnuts of *Quercus infectoria* Oliver to remove external heat. In combination with *Pinus wallichiana* A.B. Jacks. (Pinaceae), *Acorus calamus* L (Acoraceae), *Persicaria capitata* (Buch.-Ham. ex D. Don) H. Gross (Polygonaceae), *Zingiber officinale* Roscoe (Zingiberaceae), *A. naviculare* (Brühl) Stapf., and *Terminalia chebula* Retz (Combretaceae), it can be used to treat pain, abdominal distension, plague, diarrhea with mucus, and hot diarrhea. ATS is also frequently combined with its congener, *Aconitum pendulum* N. Busch (Ranunculaceae), in Tibetan medicine formulas, and the two species have a traditional ethnopharmacological basis for synergistic analgesic and anti-inflammatory effects. In modern medicine, ATS is often combined with other traditional Chinese medicines, such as Aan-Er-Ning-Ke-Li, Shi-Er-Wei-Yi-Shou-San, Er-Shi-Wu-Wei-Fei-Bing-Jiao-Nang, Shi-Yi-Wei-Jin-Se-Wan, Da-Yue-Jing-Wan, and Hong-Long-Zhen-Tong-Pian. These formulations are clinically applied to treat conditions including influenza, gastric ulcers, and Japanese encephalitis, leveraging the botanical drug’s anti-inflammatory, analgesic, and immunomodulatory effects (Zhang et al., 2012) (Figure 3). Prescriptions and proprietary Chinese medicines containing ATS are shown in Table 1.

**TABLE 1:** Chinese patent medicines and prescriptions containing *Aconitum tanguticum* (Maxim.) Stapf.

Composition	Drug names	Clinical uses	Citations
*Aconitum tanguticum* (Maxim.) Stapf (Ranunculaceae), *Gentiana straminea* Maxim. (Gentianaceae) dried flowers, *Swertia bimaculate* (Siebold & Zucc.) Hook.f. & Thomson ex C.B. Clarke (Gentianaceae), *Adhatoda vasica Ness* (Acanthaceae), *Ixeris polycephala* Cass. (Asteraceae), *Lagotis brevituba* Maxim. (Plantaginaceae), *Berberis koreana* Palib. (Berberidaceae), *Hypecoum leptocarpum* Hook.f. & Thomson (Papaveraceae), Bovis Calculus (*Bos taurus domesticus* Gmelin), *Carthamus tinctorius* L. (Asteraceae), *Herpetospermum pedunculosum* (Ser.) C.B. Clarke (Cucurbitaceae), *Chrysosplenium nudicaule* Bunge (Saxifragaceae), and *Holarrhena antidysenterica* Wall. ex G.Don (Apocynaceae)	Shi-San-Wei-Bang-Ga-San	Heat-clearing and detoxifying, cooling the liver and gallbladder. Commonly used to treat hepatobiliary disorders, including febrile, cholecystitis, and icteric hepatitis	National Pharmacopoeia Commission (2020), Peng and Ai (2021), Lian et al. (2022), Wang et al. (2024)
*Santalum album* L. (Santalaceae), *Platanus orientalis* L. (Platanaceae), Calcareous sinter, *Kaempferia galanga* L. (Zingiberaceae), *Carthamus tinctorius* L. (Asteraceae), *Vitis vinifera* L. (Vitaceae), *Swertia chirayita* (Roxb.) H. Karst. (Gentianaceae), *Glycyrrhiza uralensis* Fisch. ex DC. (Fabaceae), *Lagotis brevituba* Maxim. (Plantaginaceae), *Aconitum tanguticum* (Maxim.) Stapf (Ranunculaceae), *Hippophae rhamnoides* L. (Elaeagnaceae), *Corydalis impatiens* (Pall.) Fisch. ex DC. (Papaveraceae), *Cuminum cyminum* L.(Apiaceae), *Gentiana algida* Pall. (Gentianaceae), *Terminalia chebula* Retz. (Combretaceae), *Lancea tibetica* Hook.f. & Thomson (Mazaceae), *Terminalia bellirica* (Gaertn.) Roxb. (Combretaceae), *Eutrema scapiflorum* (Hook.f. & Thomson) Al-Shehbaz, G.Q.Hao & J.Quan Liu (Brassicaceae), *Phyllanthus emblica* L. (Phyllanthaceae), *Eremogone kansuensis* (Maxim.) Dillenb. & Kadereit (Caryophyllaceae), *Inula helenium* L. (Asteraceae), *Aconitum pendulum* N. Busch (Ranunculaceae), *Tinospora sinensis* (Lour.) Merr. (Menispermaceae), Bovis Calculus (*Bos taurus domesticus* Gmelin), and *Bergenia purpurascens* (Hook. f. & Thomson) Engl. (Saxifragaceae)	Er-Shi-Wu-Wei-Fei-Bing-Jiao-Nang	Heat-clearing and anti-inflammatory effects, promoting lung function and resolving phlegm. Treating respiratory disorders characterized by phlegm accumulation, dyspnea, fever, nasal congestion, chest discomfort, hemoptysis, and fatigue	Zhang et al. (2012)
*Bassecoia hookeri* (C.B.Clarke) V. Mayer & Ehrend. (Caprifoliaceae), *Aconitum tanguticum* (Maxim.) Stapf (Ranunculaceae), *Hypecoum erectum* L. (Papaveraceae), Bambusae Concertio Silicea (Poaceae), *Oxytropis myriophylla* (Pall.) DC.(Fabaceae), *Carthamus tinctorius* L. (Asteraceae), *Santalum album* L. (Santalaceae), *Commiphora myrrha* (T.Nees) Engl. (Burseraceae), *Dasiphora fruticosa* (L.) Rydb. (Rosaceae), Ochotonae et Trogopteri Faeces, Bovis Calculus (*Bos taurus domesticus* Gmelin), and *Moschus berezovskii* Flerov (Bovidae)	Shi-Er-Wei-Yi-Shou-San	Alleviating heat, detoxifying the body, and treating various “NIAN” diseases and plagues	Zhang et al. (2012), Ga and Jiang Yong (2023)
Bambusae Concertio Silicea (Poaceae), *Carthamus tinctorius* L. (Asteraceae), Bovis Calculus (*Bos taurus domesticus* Gmelin), *Bergenia purpuracens*, *Glycyrrhiza uralensis* Fisch. ex DC. (Fabaceae), *Cochlearia officinalis* L. (Brassicaceae), *Lagotis Brevituba* Maxim. (*Plantaginaceae*), Santalum album L. (Santalaceae), *Aconitum tanguticum* (Maxim.) Stapf (Ranunculaceae), and Sucrose	An-Er-Ning-Ke-Li	Alleviating heat and wind, eliminating phlegm, and relieving cough. It is suitable for children experiencing a cold with symptoms of wind-heat, cough with phlegm, fever, and sore throat, or an upper respiratory tract infection	Institute of Medicinal Plant Resources Development and Chinese Academy of Medical Sciences (1961), Mao (1986), Qinghai Drug Inspection Institute and Qinghai Tibetan Medicine Research Institute (1996), Zhang et al. (2012), Kuang (2019), Peng and Ai (2021), Lian et al. (2022), Ga and Jiang Yong (2023), Liu (2023), Wang et al. (2024), Wang (2024)
Draconis Os, *Swertia chirayita* (Roxb.) H.Karst.(Gentianaceae), *Carthamus tinctorius* L. (Asteraceae), *Chrysosplenium nudicaule* Bunge (Saxifragaceae), *Tanacetum tatsienense* (Bureau & Franchet) K. Bremer & Humphries (Asteraceae), *Aconitum pendulum* N. Busch (Ranunculaceae), *Aconitum tanguticum* (Maxim.) Stapf (Ranunculaceae), Bear bile powder, Sinter, *Herpetospermum pedunculosum* (Ser.) C.B. Clarke (Cucurbitaceae), *Przewalskia tangutica* Maxim. (Solanaceae), *Xanthoria elegans* (Link) Th.Fr. (Teloschistaceae), *Lagotis brachystachya* Maxim. (Plantaginaceae), *Styrax benzoin* Dryand. (Styracaceae), and Margaritifera Concha	Hong-Long-Zhen-Tong-Pian	Used to wake up the brain and relieve pain, and for migraines and angioneurotic headaches caused by cerebral stasis	Wu (2015)
*Swertia bimaculate* (Siebold & Zucc.) Hook.f. & Thomson ex C.B. Clarke (Gentianaceae), *Lagotis brevituba* Maxim. (Plantaginaceae), *Herpetospermum pedunculosum* (Ser.) C.B. Clarke (Cucurbitaceae), *Hypecoum erectum* L. (Papaveraceae), *Aconitum tanguticum* (Maxim.) Stapf (Ranunculaceae), *Berberis koreana* Palib. (Berberidaceae), *Piper pubicatulum* C.DC. (Piperaceae), and *Dolomiaea costus* (Falc.) Kasana & A.K. Pandey (Asteraceae)	Ba-Wei-Zhang-Ya-Cai-Wan	Heat-clearing and anti-inflammatory effects. It is commonly used to treat cholecystitis and early icteric hepatitis	Zhao et al. (2019)
*Terminalia chebula* Retz. (Combretaceae), Processed Sus Feces Carbonisatum, *Punica granatum* L. (Lythraceae) dried seeds, Ochotonae et Trogopteri Faeces, *Herpetospermum pedunculosum* (Ser.) C.B. Clarke (Cucurbitaceae), *Aconitum tanguticum* (Maxim.) Stapf (Ranunculaceae), *Hypecoum erectum* L. (Papaveraceae), *Embelia vestita* Roxb. (Primulaceae), *Rosa multiflora* Thunb. (Rosaceae), *Aconitum pendulum* N.Busch (Ranunculaceae), and *Moschus berezovskii* Flerov (Bovidae)	Shi-Yi-Wei-Jin-Se-Wan	Clearing heat and detoxifying, removing blood stasis. It is used to treat blood and bile flow issues in the gastrointestinal tract, gallbladder swelling, yellow discoloration of the sclera, dyspepsia, and poisoning. It has the best effect on headache, fever, and jaundice liver disease	Ministry of Health of the People’s Republic of China (1995)
*Podophyllum hexandrum* Royle (Berberidaceae), *Rubia tibetica* Hook.f (Rubiaceae), *Punica granatum* L. (Lythraceae) dried seeds, *Maharanga hookeri* (C.B.Clarke) L.Cecchi & Hilger (Boraginaceae), *Neolitsea cassia* (L.) Kosterm.(Lauraceae), *Corydalis hendersonii* Hemsl. (Papaveraceae), *Adhatoda vasica Ness* (Acanthaceae), Light Halitum, *Halite* (purple halitum), *Aconitum tanguticum* (Maxim.) Stapf (Ranunculaceae), *Inula racemosa* Hook.f. (Asteraceae), *Terminalia chebula* Retz. (Combretaceae), Dried bear bile, *Piper nigrum* L. (Piperaceae), *Mirabilis himalaica* (Edgew.) Heimerl (Nyctaginaceae), *Phyllanthus emblica* L. (Phyllanthaceae), Carne Bitis, *Kaempferia galanga* L. (Zingiberaceae), Nitroum (Potassium nitrate), Lignum Dalbergiae Odoriferae, Fructus Hippophae, *Aquilaria malaccensis* Lam. (Thymelaeaceae), Cinnabaris, *Myristica fragrans* Houtt. (Myristicaceae), *Lycium barbarum* L. (Solanaceae), Lacca, and *Coriandrum sativum* L. (Apiaceae)	Er-Shi-Wu-Wei-Gui-Jiu-Wan	Expelling wind and relieving pain, regulating menstrual blood, treating women’s blood syndrome, wind syndrome, uterine worm disease, upset blood deficiency, irregular menstruation, and pain in the lower limb joints, lower abdomen, liver, gallbladder, and upper body	Guo et al. (2019), Zhang et al. (2023), Zhao et al. (2023)
Calcareous sinter, *Santalum album* L. (Santalaceae), *Lagotis brevituba* Maxim. (Plantaginaceae), *Aconitum tanguticum* (Maxim.) Stapf (Ranunculaceae), *Vallisneria natans* (Lour.) H. Hara (Hydrocharitaceae), *Carthamus tinctorius* L. (Asteraceae), *Cochlearia officinalis* L. (Brassicaceae), *Cochlearia officinalis* L. (Brassicaceae), and Bovis Calculus (*Bos taurus domesticus* Gmelin)	Jiu-Wei Shi-Hui-Hua-San	Clearing heat, detoxifying, relieving cough, and calming nerves; also used to treat pediatric pneumonia, high fever, irritability, and cough	Liu et al. (2021), Yu (2023)
*Gypsum rubrum* L., Bambusae Concertio Silicea (Poaceae), *Carthamus tinctorius* L. (Asteraceae), *Myristica fragrans* Houtt. (Myristicaceae), *Lanxangia tsao-ko* (Crevost & Lemarié) M.F. Newman & Škorničk.(Zingiberaceae), *Amomum kravanh* Pierre ex Gagnep. (Zingiberaceae), *Syringa oblata* Lindl. (Oleaceae), *Terminalia chebula* Retz. (Combretaceae), *Phyllanthus emblica* L. (Phyllanthaceae), *Santalum album* L. (Santalaceae), *Holarrhena antidysenterica* Wall. ex G.Don (Apocynaceae), *Herpetospermum pedunculosum* (Ser.) C.B. Clarke (Cucurbitaceae), *Punica granatum* L. (Lythraceae) dried seeds, *Inula racemosa* Hook.f. (Asteraceae), Benzonium, Ochotonae et Trogopteri Faeces, *Aconitum tanguticum* (Maxim.) Stapf (Ranunculaceae), *Swertia bimaculate* (Siebold & Zucc.) Hook.f. & Thomson ex C.B. Clarke (Gentianaceae), etc.	Da-Yue-Jing-Wan	Clearing heat and detoxifying, promoting digestion and relieving distention; treating poisoning; clearing hidden heat, old heat, fluctuating heat, dyspepsia, acute abdominal pain, worm disease, yellow water disease, fullness, and other complications	Cai Xiang (2020)

## 5 Chemical composition

### 5.1 Alkaloids

**FIGURE 4:**
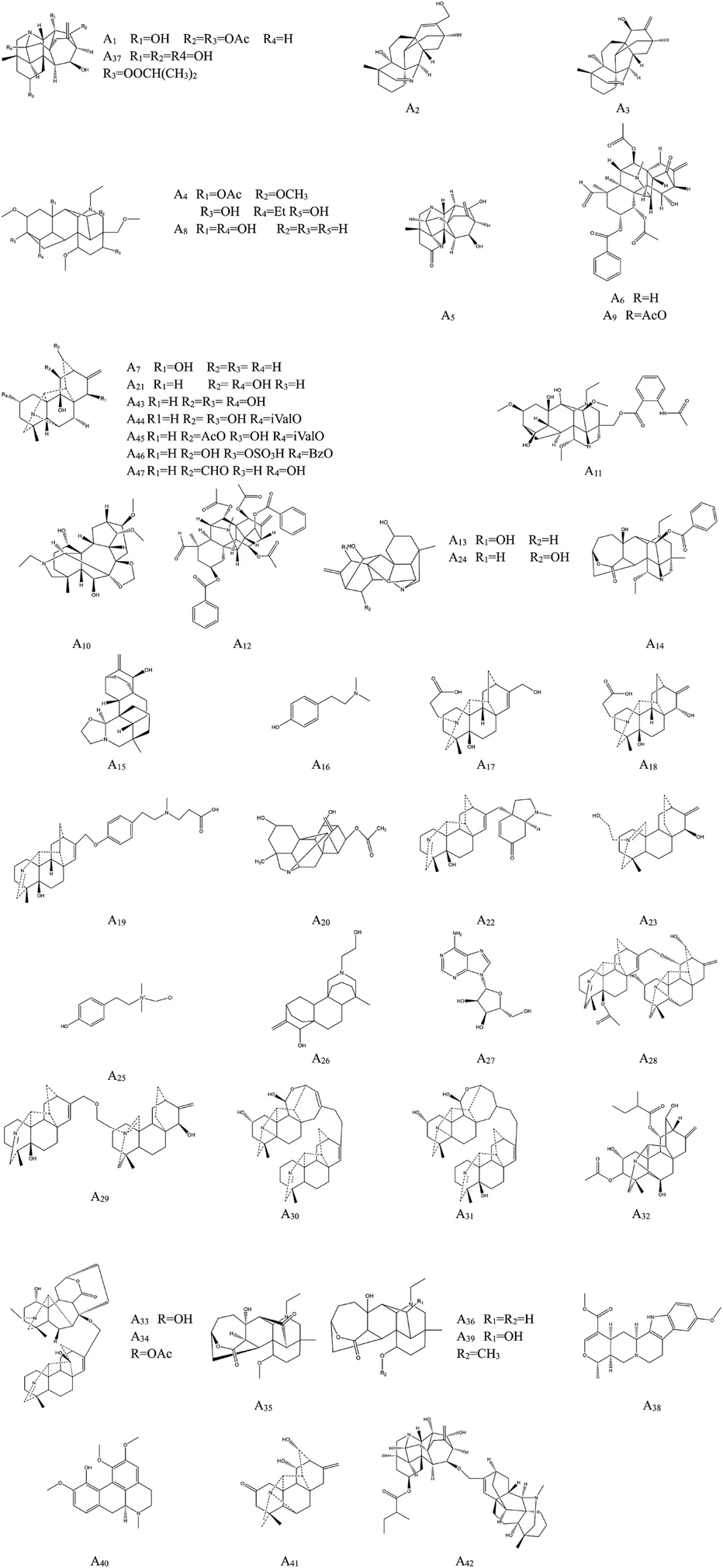
Structures of the alkaloids in *Aconitum tanguticum* (Maxim.) Stapf.

ATS is primarily composed of alkaloids, with diterpene alkaloids being the main research focus. In analyses of ATS, Zhang (1990) identified Guan-fu base A, and Wu (2015) found naviculine A, naviculine B, and aconitine. Its unique components include trichocarpine A/B, dehydroheteratisine, heterophyllidine, heteratisine, and benzoylheteratisine, all of which belong to the lactone alkaloid class. The alkaloid components of ATS are shown in Figure 4 and Table 2.

**TABLE 2:** Alkaloid components of *Aconitum tanguticum* (Maxim.) Stapf.

Compound	Chemical name	Molecular weight	Formula	Type	Citations
A_1_	Guan-fu base A	429.51	C_24_H_31_NO_6_	C20-type diterpenoid alkaloid	Zhang (1990), Li (2008), Wu (2015)
A_2_	Naviculine A	313.44	C_20_H_27_NO_2_	C20-type diterpenoid alkaloid	Wu (2015)
A_3_	Naviculine B	313.44	C_20_H_27_NO_2_	C20-type diterpenoid alkaloid	Wang et al. (2002), Lin et al. (2009), Zhang et al. (2013), Yang et al. (2016b), Wu (2015)
A_4_	Aconitine	645.75	C_34_H_47_NO_11_	C19-type diterpenoid alkaloid	Wu (2015)
A_5_	Hetisinone	327.42	C_20_H_25_NO_3_	C20-type diterpenoid alkaloid	Zhang (1990), Ministry of Health of the People’s Republic of China (1995), Li (2008), Wang et al. (2002), Wu (2015), Guo et al. (2019), Zhao et al. (2019), Cai Xiang (2020), Qu et al. (2011), Liu et al. (2021), Yu (2023), Zhang et al. (2023), Zhao et al. (2023)
A_6_	Barbisine	577.63	C_33_H_35_NO_9_	C20-type diterpenoid alkaloid	Wu (2015)
A_7_	9-Hydroxynominine	313.44	C_20_H_27_NO_2_	C19-type diterpenoid alkaloid	Wu (2015), Qu et al. (2011)
A_8_	Talatizamine	421.58	C_24_H_39_NO_5_	C19-type diterpenoid alkaloid	Wang et al. (2002), Wu (2015)
A_9_	Tangutisine B	577.63	C_33_H_35_NO_9_	C20-type diterpenoid alkaloid	Wu (2015), Wang et al. (2005)
A_10_	Pacidine	435.56	C_24_H_37_NO_6_	C19-type diterpenoid alkaloid	Wang et al. (2005)
A_11_	14-Deacetylajadine	614.74	C_33_H_46_N_2_O_9_	C19-type diterpenoid alkaloid	Wang et al. (2005)
A_12_	Tangutisine A	725.79	C_41_H_43_NO_11_	C19-type diterpenoid alkaloid	Wu (2015), Li et al. (2004)
A_13_	Tanwusine	329.44	C_20_H_27_NO_3_	C19-type diterpenoid alkaloid	Chen and Song (1985)
A_14_	Benzoylheratisine	495.62	C_29_H_37_NO_6_	C19-type diterpenoid alkaloid	Yang et al. (2016b), Ling and Chen (2009), Xu et al. (2013), Qing Cai et al. (2004), He et al. (2014) Chen and Song (1985)
A_15_	Atisine	343.51	C_22_H_33_NO_2_	C20-type diterpenoid alkaloid	Wang et al. (2002), Wu (2015), Joshi et al. (2001), Qu et al. (2011), Chen et al. (2021), Chen and Song (1985)
A_16_	Hordenine	165.24	C_10_H_15_NO	Simple phenethylamine alkaloid	Yang (2016), Wang et al. (2002), Wu (2015), Joshi et al. (2001), Chen and Song (1985)
A_17_	Tangutidine A	387.52	C_23_H_33_NO_4_	C20-type diterpenoid alkaloid	Li et al. (2021)
A_18_	Tangutidine B	387.52	C_23_H_33_NO_4_	C20-type diterpenoid alkaloid	Li et al. (2021)
A_19_	Tangutidine C	518.70	C_32_H_42_N_2_O_4_	C20-type diterpenoid alkaloid	Li et al. (2021)
A_20_	11-Acetylhetisine	371.47	C_22_H_29_NO_4_	C20-type diterpenoid alkaloid	Yang (2016)
A_21_	2β,9β,11β,13β-Tetrahydrohetisine	345.44	C_20_H_27_NO_4_	C19-type diterpenoid alkaloid	Yang (2016)
A_22_	Tanaconitine	446.63	C_29_H_38_N_2_O_2_	C20-type diterpenoid alkaloid	Yang (2016), Xu et al. (2013), Qu et al. (2011), Yang et al. (2016a), Qu et al. (2011)
A_23_	Atenesine hydrochloride	344.52	C_22_H_34_NO_2_	C20-type diterpenoid alkaloid	Yang (2016)
A_24_	Hetisine	329.44	C_20_H_27_NO_3_	C19-type diterpenoid alkaloid	Yang (2016), Yang et al. (2016a)
A_25_	Candicine chloride	214.71	C_11_H_17_NO^+^Cl	Simple phenethylamine alkaloid	Yang (2016)
A_26_	Dihydroatisine	345.5	C_22_H_35_NO_2_	C20-type diterpenoid alkaloid	Yang (2016), Yang et al. (2016a)
A_27_	Adenosine	267.25	C_10_H_13_N_5_O_4_	Nucleoside alkaloid	Yang (2016), Li et al. (2014)
A_28_	Tanguticinine F	666.90	C_42_H_54_N_2_O_5_	C20-type diterpenoid alkaloid	Bai (2022), Ye et al. (2023)
A_29_	Tanguticinine G	639.94	C_42_H_59_N_2_O_3_	C20-type diterpenoid alkaloid	Bai (2022), Ye et al. (2023)
A_30_	5-Deoxyanthoroidine B	610.88	C_40_H_54_N_2_O_3_	C20-type diterpenoid alkaloid	Bai (2022)
A_31_	Anthoroidine B	626.88	C_40_H_54_N_2_O_4_	C20-type diterpenoid alkaloid	Yang et al. (2021), Bai (2022)
A_32_	Geyerinine	487.59	C_27_H_37_NO_7_	C20-type diterpenoid alkaloid	Lin et al. (2009), Zhang et al. (2013)
A_33_	Trichocarpine B	673.42	C_41_H_57_N_2_O_6_	C20-type diterpenoid alkaloid	Lin et al. (2009), Zhang et al. (2013)
A_34_	Trichocarpine A	715.43	C_43_H_58_N_2_O_7_	C20-type diterpenoid alkaloid	Lin et al. (2009), Zhang et al. (2013)
A_35_	Dehydroheteratisine	389.49	C_22_H_31_NO_5_	C19-type diterpenoid alkaloid	Yang et al. (2016b), Lin et al. (2009), Ling and Chen (2009), Zhang et al. (2013), Wu (2015)
A_36_	Heterophyllidine	389.54	C_23_H_35_NO_4_	C19-type diterpenoid alkaloid	Yang et al. (2016b), Lin et al. (2009), Ling and Chen (2009), Zhang et al. (2013), Wu (2015)
A_37_	Guan-fu base Z	415.53	C_24_H_33_NO_5_	C20-type diterpenoid alkaloid	Yang et al. (2016b), Lin et al. (2009), Ling and Chen (2009), Zhang et al. (2013)
A_38_	Heterophylline	382.46	C_22_H_26_N_2_O_4_	Simple indole alkaloid	Yang et al. (2016b), Lin et al. (2009), Wu (2015)
A_39_	Heteratisine	391.51	C_22_H_33_NO_5_	C19-type diterpenoid alkaloid	Yang (2016), Xu et al. (2013), Yang et al. (2016a), Desai and Pelletier (1993), Li et al. (2014), Qing Cai et al. (2004), Wu (2015), Joshi et al. (2001), Chen and Song (1985)
A_40_	Isocorydine	341.41	C_20_H_23_NO_4_	Simple isoquinoline alkaloid	He et al. (2014), Yin and Jiang (2023)
A_41_	11α,13α-Dihydroxyhetisinone	327.42	C_20_H_25_NO_3_	C20-type diterpenoid alkaloid	Yang (2016), Bessonova and Saidkhodzhaeva (2000), Yang et al. (2016a)
A_42_	Trichocarpine	740.48	C_46_H_64_N_2_O_6_	C20-type diterpenoid alkaloid	Lin et al. (2010), Zhang et al. (2013)
A_43_	Tanguticuline A	346.20	C_20_H_27_NO_4_	C19-type diterpenoid alkaloid	Fan et al. (2019)
A_44_	Tanguticuline B	430.26	C_25_H_35_NO_5_	C19-type diterpenoid alkaloid	Fan et al. (2019)
A_45_	Tanguticuline C	472.27	C_27_H_37_NO_6_	C19-type diterpenoid alkaloid	Fan et al. (2019)
A_46_	Tanguticuline D	530.19	C_27_H_31_NO_8_S	C19-type diterpenoid alkaloid	Fan et al. (2019)
A_47_	Tanguticuline E	344.19	C_20_H_25_NO_4_	C19-type diterpenoid alkaloid	Fan et al. (2019)

### 5.2 Volatile oils

**FIGURE 5:**
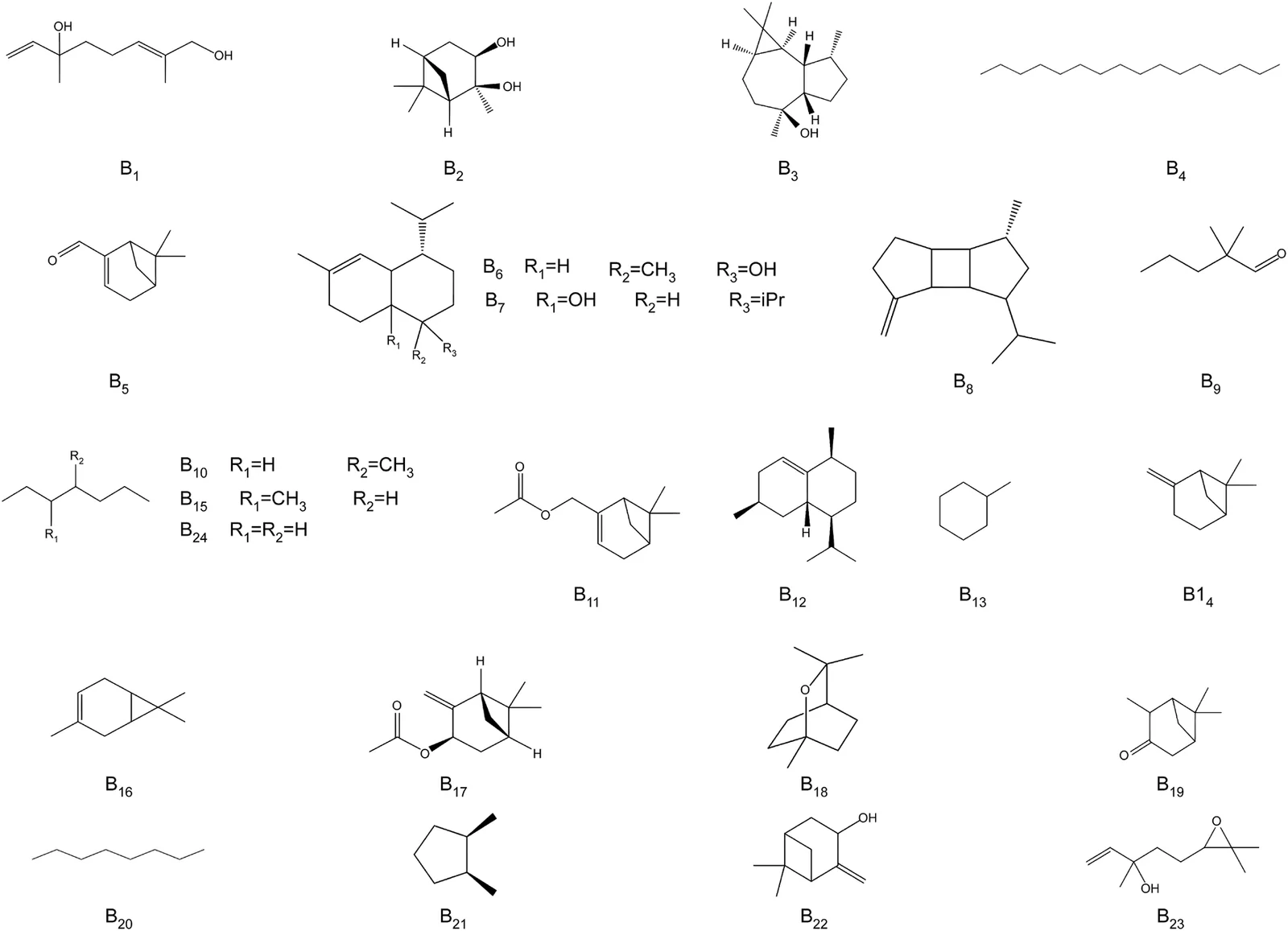
Structures of the volatile oil in *Aconitum tanguticum* (Maxim.) Stapf.

The essential oil of ATS is a complex mixture of volatile compounds. A previous study reported the extraction of the essential oils from ATS using steam distillation and its analysis using gas chromatography–mass spectrometry (Zhang et al., 2009). The main components of volatile oil were (-)-trans pinocarvyl acetate, cineole, and 3-pinanone. These are the representative volatile oil constituents of ATS. The volatile oil composition of ATS is shown in Figure 5 and Table 3.

**TABLE 3:** Volatile oil components of *Aconitum tanguticum* (Maxim.) Stapf (Zhang et al., 2009).

Compound	Chemical name	Molecular weight	Formula
B_1_	8-Hydroxylinalool	170.25	C_10_H_18_O_2_
B_2_	(+)-Pinanediol	170.25	C_10_H_18_O_2_
B_3_	Viridiflorol	222.37	C_15_H_26_O
B_4_	Hexadecane	226.45	C_16_H_34_
B_5_	6,6-Dimethyl-2-norpinene-2-carboxaldehyde	150.22	C_10_H_14_O
B_6_	Cadinol	222.37	C_15_H_26_O
B_7_	(-)-Cubenol	222.37	C_15_H_26_O
B_8_	Decahydro-3α-methyl-6-methylene-1-(1-methylethyl)-cyclobuta [1,2:3,4]dicyclopentene	204.36	C_15_H_24_
B_9_	2,2-Dimethyl-pentanal	114.19	C_7_H_14_O
B_10_	4-Methyl-heptane	114.23	C_8_H_18_
B_11_	(-)-Myrtenyl acetate	194.27	C_12_H_18_O_2_
B_12_	Cadinene	206.37	C_15_H_26_
B_13_	Methyl-cyclohexane	98.19	C_7_H_14_
B_14_	β-pinene	136.24	C_10_H_16_
B_15_	3-Methylheptane	114.23	C_8_H_18_
B_16_	3-Carene	136.24	C_10_H_16_
B_17_	(-)-Tran-pinecarvyl acetate	194.27	C_12_H_18_O_2_
B_18_	Cineole	154.25	C_10_H_18_O
B_19_	Pinocamphone	152.24	C_10_H_16_O
B_20_	Octane	114.23	C_8_H_18_
B_21_	Cis-1,2-dimethylcyclopentane	98.19	C_7_H_14_
B_22_	Pinocarveol	152.24	C_10_H_16_O
B_23_	Linalool oxide	170.25	C_10_H_18_O_2_
B_24_	Heptane	100.20	C_7_H_16_

### 5.3 Flavonoids

**FIGURE 6:**
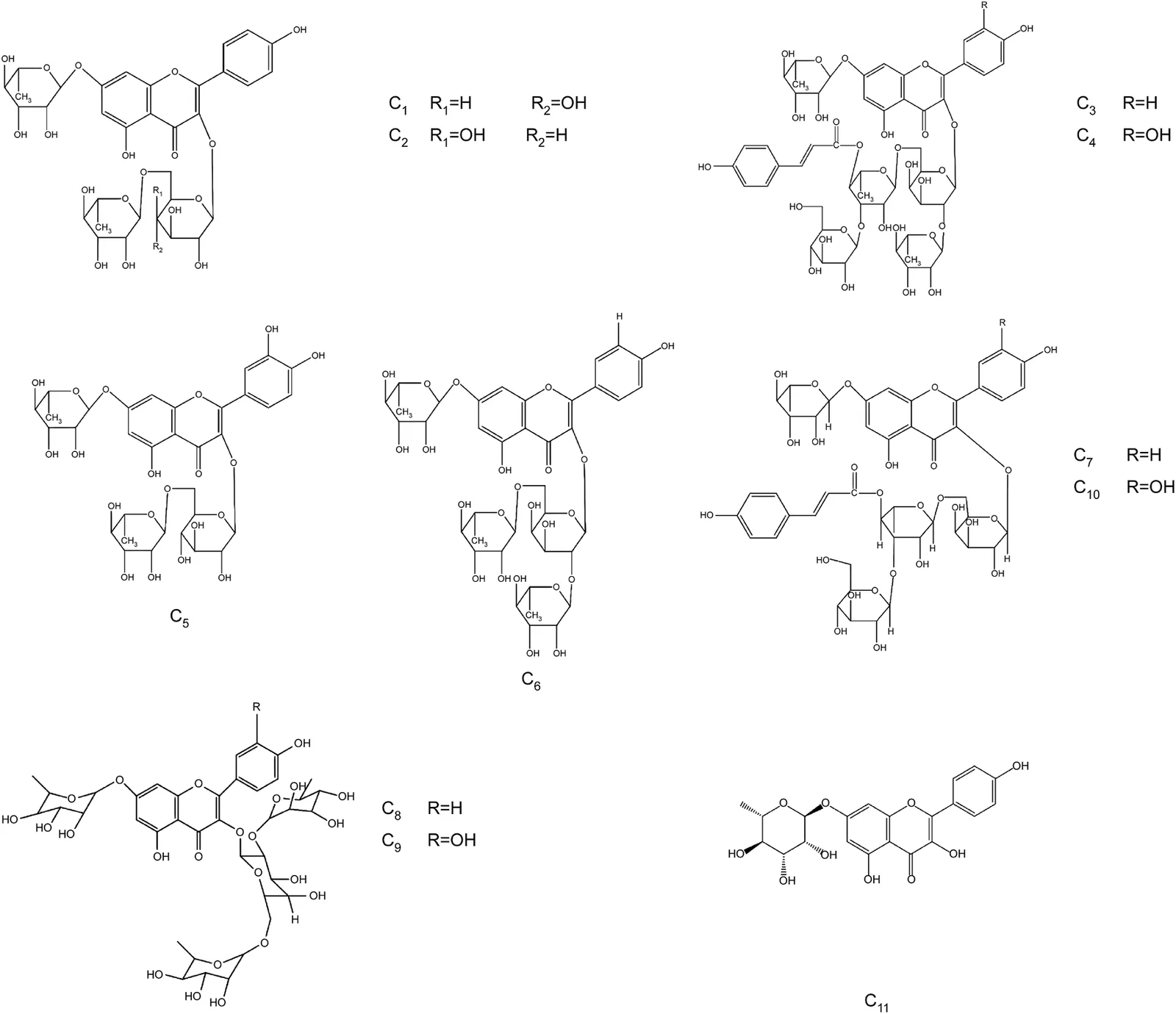
Structures of the flavonoids in *Aconitum tanguticum* (Maxim.) Stapf.

ATS contains various flavonoid glycosides, such as reported kaempferol glycosides and quercetin glycosides, which exert auxiliary antioxidant and anti-inflammatory activities. The flavonoid composition of ATS is displayed in Figure 6 and Table 4.

**TABLE 4:** Flavonoid components of *Aconitum tanguticum* (Maxim.) Stapf.

Compound	Chemical name	Molecular weight	Formula	Citations
C_1_	Kaempferol-3-O-[α-L-rhamnopyranosyl-(1→6)-β-D-glucopyranoside]-7-O-α-L-rhamnopyranoside	740.66	C_33_H_40_O_19_	Yang (2016)
C_2_	Kaempferol-3-O-[α-L-rhamnopyranosyl-(1→6)-β-D-galactopyranoside]-7-O-α-L-rhamnopyranoside	740.66	C_33_H_40_O_19_	Yang (2016)
C_3_	Kaempferol-3-O-α-L-rhamnopyranosyl-(1→2)-[β-D-glucopyranosyl-(1→3)-α-L-(4-O-trans-p-coumaroyl-rhamnopyranosyl (1→6)]-β-D-galactopyranoside-7-O-α-L-rhamnopyranosid	1195.09	C_54_H_66_O_30_	Yang (2016)
C_4_	Quercetin-3-O-α-L-rhamnopyranosyl-(1→2)-[β-D-glucopyranosyl-(1→3)-α-L-(4-O-trans-p-coumaroyl-rhamnopyranosyl)-(1→6)]-β-D-galactopyranoside-7-O-α-L-rhamnopyranoside	1211.09	C_54_H_66_O_31_	Yang (2016)
C_5_	Quercetin-3-O-α-L-rhamnopyranosyl-(1→6)-β-D-glucopyranoside-7-O-α-L-rhamnopyranoside	756.66	C_33_H_40_O_20_	Yang (2016), Ling and Chen (2009)
C_6_	Kaempferol-3-O-α-L-rhamnopyranosyl-(1→2)-[α-L-rhamnopyranosyl-(1→6)]-β-D-galactopyranoside-7-O-α-L-rhamnopyranoside	886.81	C_39_H_50_O_23_	Yang (2016), Ling and Chen (2009)
C_7_	Kaempferol-3-O-[β-D-glucopyranosyl-(1→3)-(4-otrans-p-coumaroyl)]-α-L-rhamnopyranosyl-(1→6)-β-D-galactopyranside-7-O-α-L-rhamnopyranoside	1048.95	C_48_H_56_O_26_	Yang (2016), Xu et al. (2013), Ling and Chen (2009)
C_8_	Kaempferol 3-O-α-L-rhamnopyranosyl-(1→2)-[α-L-rhamnopyranosyl-(1→6)]-β-D-galactopyranside-7-O-α-L-rhamnopyranoside	886.81	C_39_H_50_O_23_	Yang (2016), Xu et al. (2013), Ling and Chen (2009)
C_9_	Quercetin 3-O-α-L-rhamnopyranosyl-(1→2)-[α-L-rhamnopyranosyl-(1→6)]-β-D-galactopyranside-7-O-α-L-rhamnopyranoside	902.80	C_39_H_50_O_24_	Yang (2016), Xu et al. (2013)
C_10_	Quercetin 3-O-[β-D-glucopyranosyl-(1→3)-(4-O-trans-p-coumaroyl)]-α-L-rhamnopyranosyl-(1→6)-β-D-galactopyranoside-7-O-α-L-rhamnopyranoside	1064.95	C_48_H_56_O_27_	Yang (2016), Xu et al. (2013), Ling and Chen (2009)
C_11_	Kaempferol-7-O-α-L-rhamnopyranoside	432.38	C_21_H_2_O_10_	Yang (2016), Luo et al. (2012)

### 5.4 Phenolic acid and its derivatives

**FIGURE 7:**
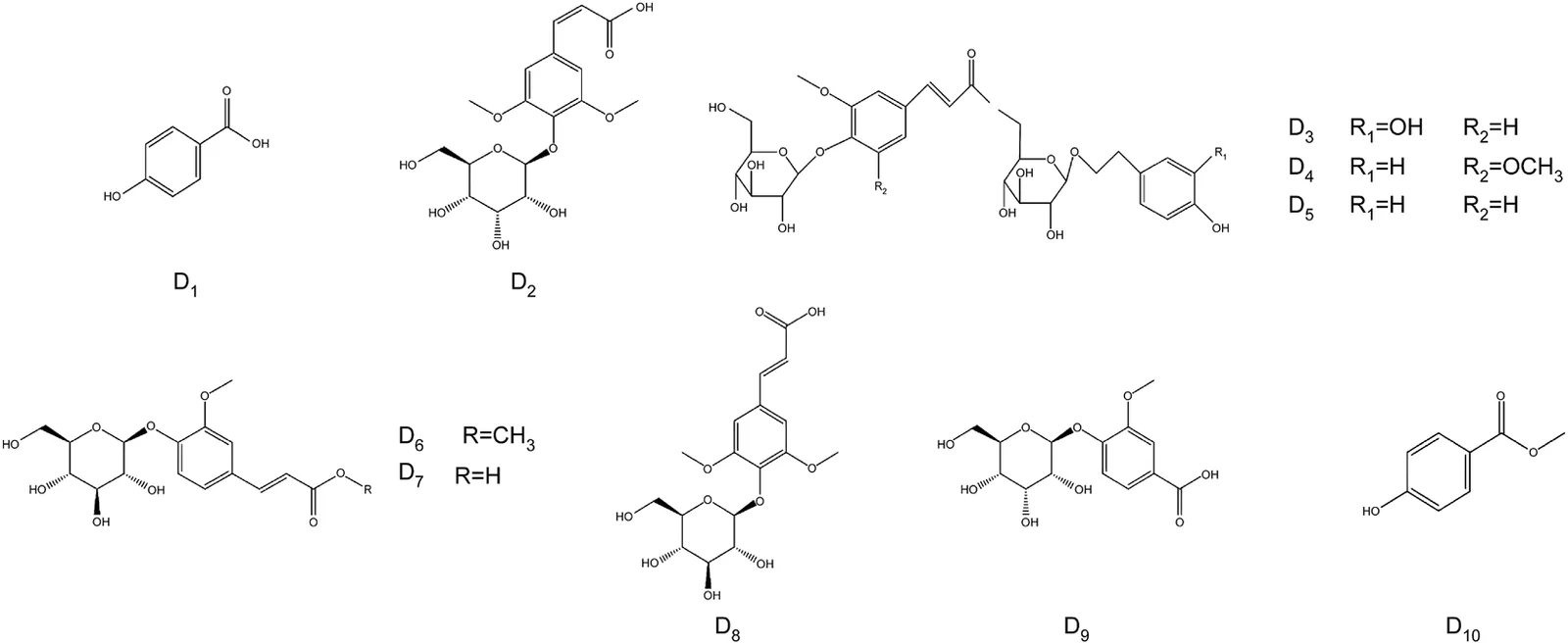
Structures of the phenolic acids and their derivatives in *Aconitum tanguticum* (Maxim.) Stapf.

Phenolic acids and their derivatives include complex phenolic acid ester glycosides (e.g., 3,4-dihydroxyphenethoxy-8-O-β-D-[6-O-(4-O-β-D-glucopyranosyl)-feruloyl]glucopyranoside), phenolic acid glycosides (e.g., (Z)-sinapic acid-4-O-β-D-allopyranoside), and simple phenolic acids and their derivatives (e.g., p-hydroxybenzoic acid). These phenolic acids are common secondary metabolites isolated and identified from ATS. The composition of phenolic acids and their derivatives in ATS is shown in Figure 7 and Table 5.

**TABLE 5:** Phenolic acid and its derivatives in *Aconitum tanguticum* (Maxim.) Stapf.

Compound	Chemical name	Molecular weight	Formula	Citations
D_1_	P-Hydroxybenzoic acid	138.12	C_7_H_6_O_3_	Yang (2016)
D_2_	(Z)-Sinapicacid-4-O-β-D-allopyranoside	386.35	C_17_H_22_O_10_	Yang (2016)
D_3_	3,4-Dihydroxyphenethoxy-8-O-β-D-[6-O-(4-O-β-D-glucopyranosyl)-feruloyl] glucopyranoside	654.62	C_30_H_38_O_16_	Yang (2016)
D_4_	4-Hydroxyphenethoxy-8-O-β-D-[6-O-(4-O-β-D-glucopyranosyl)-sinapoyl] glucopyranoside	668.64	C_31_H_60_O_16_	Yang (2016)
D_5_	4-Dihydroxyphenethoxy-8-O-β-D-[6-O-(4-O-β-D-glucopyranosyl)-feruloyl] glucopyranoside	638.62	C_30_H_38_O_15_	Yang (2016)
D_6_	4-O-β-D-Glucopyranosyl-(E)-ferulic acid methyl ester	370.35	C_17_H_22_O_9_	Yang (2016)
D_7_	(E)-Ferulic-acid-4-O-β-D-glucopyranoside	356.33	C_16_H_2_0O_9_	Yang (2016), Xu et al. (2013)
D_8_	(E)-Sinapic acid-4-O-β-D-glucopyranoside	386.35	C_17_H_22_O_10_	Yang (2016), Xu et al. (2013)
D_9_	Vanillic acid-4-O-β-D-allopyranoside	300.29	C_14_H_18_O_9_	Yang (2016), Xu et al. (2013)
D_10_	P-Hydroxybenzoic acid methyl ester	152.15	C_8_H_8_O_3_	Yang (2016), Li et al. (2014)

### 5.5 Other components

**FIGURE 8:**
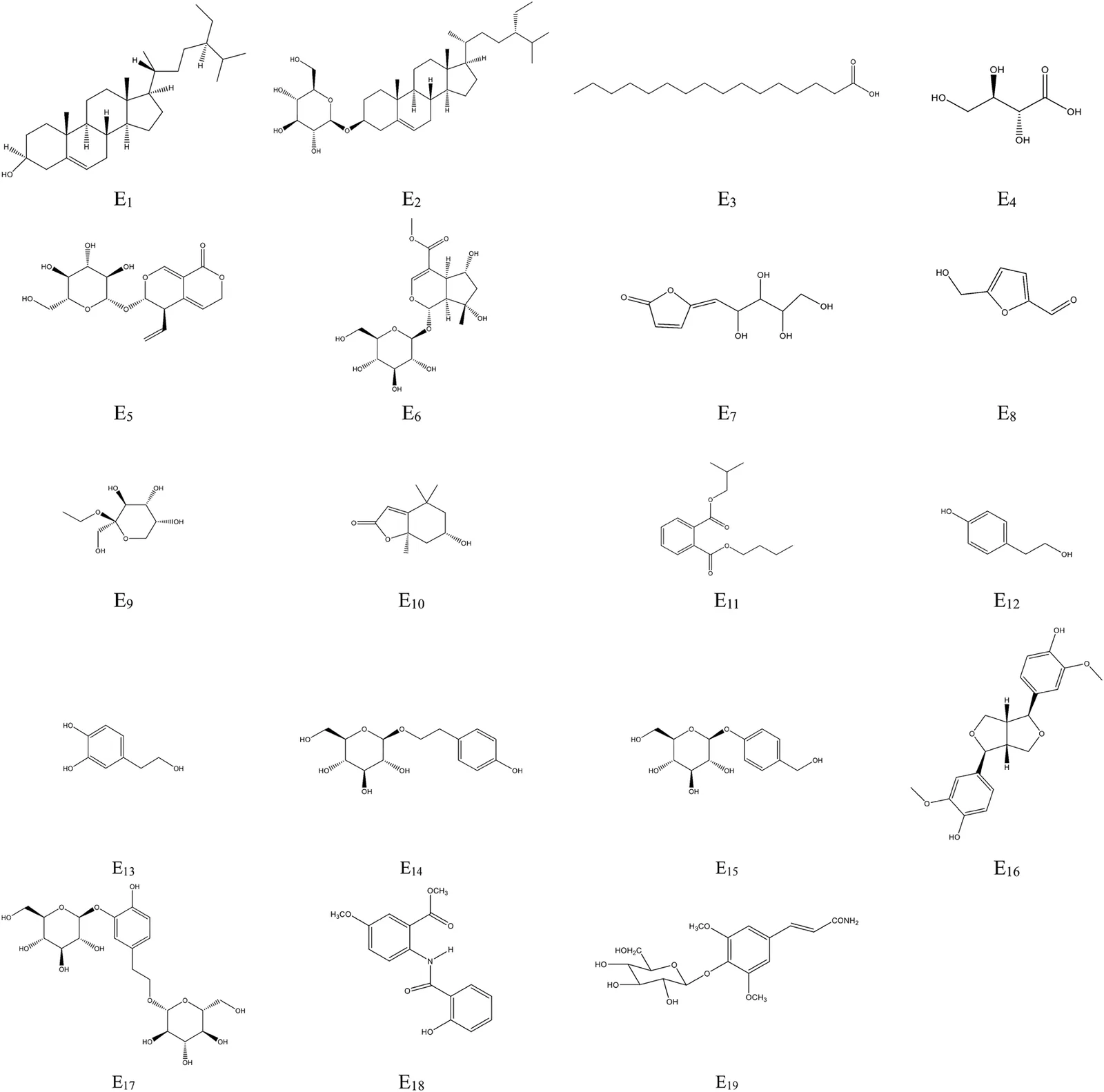
Structures of the other classes of compounds in *Aconitum tanguticum* (Maxim.) Stapf.

ATS contains numerous trace elements, with their content in the following order: Fe > Cu > Zn > Cr > Pb (Yang, 2017). ATS also contains various other components, such as β-sitosterol, daucosterol, and gentiopicroside, as presented in Figure 8 and Table 6.

**TABLE 6:** Other components in *Aconitum tanguticum* (Maxim.) Stapf.

Compound	Chemical name	Molecular weight	Formula	Citations
E_1_	β-Sitosterol	414.72	C_29_H_50_O	Zhang (1990), Yang (2016), Li (2008), Yang et al. (2016a)
E_2_	Daucosterol	576.86	C_35_H_60_O_6_	Zhang (1990), Yang (2016), Li (2008)
E_3_	Palmitic acid	256.43	C_16_H_32_O_2_	Zhang (1990), Yang (2016), Li (2008)
E_4_	(2R,3R)-2,3,4-trihydroxybutyric acid	136	C_4_H_8_O_5_	Yang (2016)
E_5_	Gentiopicroside	356.33	C_16_H_2_0O_9_	Yang (2016)
E_6_	Shanzhiside methyl ester	406.38	C_17_H_26_O_11_	Yang (2016)
E_7_	Icariside B_1_	216.19	C_9_H_12_O_6_	Yang (2016)
E_8_	5-Hydroxymethyl furfural	126.11	C_6_H_6_O_3_	Yang (2016)
E_9_	Ethyl-β-D-fructopyranoside	208.21	C_8_H_16_O_6_	Yang (2016)
E_10_	Loliolide	196.25	C_11_H_16_O_3_	Yang (2016)
E_11_	Phthalic acid butyl ester	278.35	C_26_H_22_O_4_	Yang (2016), Yang et al. (2016a)
E_12_	P-Hydroxyphenylethanol	138.17	C_8_H_10_O_2_	Yang (2016)
E_13_	3,4-Dihydroxy phenylethanol	154.16	C_8_H_10_O_3_	Yang (2016), Li et al. (2014)
E_14_	Salidroside	300.31	C_14_H_2_0O_7_	Yang (2016), Li et al. (2014)
E_15_	Gastrodin	286.28	C_13_H_18_O_7_	Yang (2016), Li et al. (2014)
E_16_	(+)-Pinoresinol	358.39	C_20_H_22_O_6_	Yang (2016)
E_17_	2-(3-β-D-Glucopyranosyloxy-4-hydroxyphenyl)-ethanol-1-O-β-D-glucopyranoside	478.45	C_20_H_30_O_13_	Yang (2016)
E_18_	5-Methoxy-N-salicylanthranilic acid methyl ester	302.1	C_16_H_15_NO_5_	Fan et al. (2019)
E_19_	3,5-Dimethoxy-4-hydroxycinnamamide-4-O-β-D-glucopyranoside	384.13	C_17_H_23_NO_9_	Fan et al. (2019)

## 6 Pharmacological effects

### 6.1 Anti-inflammatory effects

Modern pharmacological studies have systematically demonstrated the anti-inflammatory and analgesic properties of ATS and its active compounds, particularly its alkaloids, across multiple experimental models.

Zeng et al. (2009) reported that total alkaloids isolated from Bangga [ATS and *A. naviculare* (Brühl) Stapf. (Ranunculaceae)] with a purity exceeding 75% significantly inhibited adjuvant-induced arthritis in rats. The rats were divided into blank control (treated with normal saline), model, aspirin (20 mg/kg), low-dose total alkaloids (200 mg/kg), and high-dose total alkaloids (500 mg/kg) groups. Cyclooxygenase-2 (COX-2), interleukin-1 (IL-1), tumor necrosis factor (TNF), and prostaglandin E_2_ (PGE_2_) levels were determined by radioimmunoassay. The total alkaloids reduced these inflammatory factors in a dose-dependent manner. In another *in vitro* enzyme activity inhibition assay, recombinant COX-2 protein expressed in Sf9 insect cells was used to screen enzyme activity in blank, positive control (celecoxib, 1.0 mmol/L), and high- and low-dose total alkaloids groups (1 and 0.2 mg/L). All alkaloid-treated groups significantly inhibited COX-2 activity (P < 0.01), providing enzymatic evidence for the anti-inflammatory effect of Bangga alkaloids.

Qu (2009) and Wu et al. (2017) further confirmed their dose-dependent anti-inflammatory and analgesic effects in multiple animal models. Qu (2009) evaluated total alkaloids with a purity exceeding 75% in a series of acute inflammatory models, including xylene-induced ear edema, acetic acid-induced increase in abdominal capillary permeability, acetic acid-induced writhing reaction, carrageenan-induced, and zymosan A-induced paw edema. Normal saline was used for the blank control group, while the low-dose and high-dose groups were administrated total alkaloids at 0.2 g/kg and 0.4 g/kg, respectively. Aspirin (0.02 g/kg) was used in the xylene and acetic acid-related models, while indomethacin was applied in carrageenan-induced (0.017 g/kg) and zymosan A-induced paw edema (10 mg/kg) models. Both total alkaloid doses could significantly relieve inflammatory swelling, reduce vascular exudation, and alleviate pain responses (P < 0.05, P < 0.01) in a dose-dependent manner. Notably, the high-dose group exerted better efficacy than positive control drugs in the zymosan A-induced inflammatory model.

Wu et al. (2017) examined the anti-inflammatory and analgesic activities of ethanol-extracted total alkaloids from ATS with a content of 0.3323%. The experimental models contained xylene-induced ear edema, subcutaneous agar granuloma hyperplasia, and acetic acid-induced writhing test. The blank control group was given equal-volume solvent, the positive control groups were treated with prednisone acetate (45 mg/kg) for anti-inflammatory experiments and lappaconitine hydrobromide (1.0 mg/kg) for analgesic experiments, and the experimental groups were set at doses of 100 mg/kg, 75 mg/kg, and 50 mg/kg. High and medium doses presented obvious inhibitory effects on acute inflammation and chemical pain (P < 0.05, P < 0.01). Only the high-dose group produced weak intervention effects on chronic proliferative inflammation, while the low dose had no obvious pharmacological activity. These findings demonstrated that the total alkaloids of ATS possessed definite anti-inflammatory and analgesic pharmacological activities.

Collectively, these studies have verified the anti-inflammatory activity of total alkaloids from Bangga; however, several limitations remain. Zeng et al. (2009) failed to distinguish its two original plant sources, ATS and *A. naviculare* (Brühl.) Stapf, and only focused on COX-2 enzyme activity, resulting in a one-sided exploration of anti-inflammatory mechanisms. Additionally, insufficient *in vivo* evaluation indicators and simple dose gradients were adopted, and the active metabolites were not identified. Similarly, Qu (2009) did not differentiate the botanical origins of the medicinal material and merely performed pharmacological experiments on animal models without in-depth exploration of molecular mechanisms, with a restricted dose design. While Wu et al. (2017) clarified the botanical origin of Bangga, their study lacked investigations on molecular mechanisms and identification of active metabolites, the efficacy evaluation mainly relied on macroscopic indicators without quantitative data such as inflammatory factors, and the applied dose gradients failed to determine the optimal dosage. Future research should focus on botanical origin identification, active component analysis, mechanism elucidation, and dosage optimization.

The *in vivo* studies described above confirm that the total alkaloids of ATS possess significant anti-inflammatory and analgesic activities. Subsequent molecular mechanism research further revealed that its anti-inflammatory effect is closely related to the regulation of the phosphoinositide 3-kinase- protein kinase B (PI3K-AKT) signaling pathway (Hachul et al., 2021; Harikrishnan et al., 2018; Kim et al., 2018; Wang et al., 2019). Additional active ingredients, core targets, and related signaling pathways are summarized in Table 7. Molecular docking analyses further indicate that the key active components of ATS exhibit high binding affinity to Akt serine/threonine kinase (AKT1) and Janus kinase 2 (JAK2), two central targets within this pathway (Bai et al., 2023). Overall, ATS exerts multi-level anti-inflammatory and analgesic effects, including direct COX-2 inhibition and upstream regulation of inflammatory signaling via the PI3K-AKT axis, providing scientific support for its traditional use and guiding future research directions.

**TABLE 7:** Major active components, core targets, and their associated signaling pathways.

Compound	Active constituent	Related targets	Signaling pathways	Citations
1	6-Benzoy lheteratisine	AKT1, JAK2, TNF, MAPK3, MAPK1, SRC, HSP90AA1, STAT3, PIK3CA, NF-κB, IL-1β, Keap1	PI3K-Akt pathway, MAIK pathway, NF-κB pathway, HIF-1 pathway, TGF-β/Smad pathway, Keap1/Nrf2/HO-1 pathway	Bai et al. (2023), Meng et al. (2025), Cui et al. (2024), and Ye et al. (2023)
2	Guan-Fu Base A			
3	Atisine			
4	Heteratisine			
5	Tangutisine A			
6	Hetisinone			
7	Hordenine			
8	Tangutisine B			
9	Talatizamine			
10	Dehydroheteratisine			
11	Heterophyllidine			
12	Heterophylline			
13	Venulol			
14	Barbisine			
15	Naviculine A			
16	Naviculine B			
17	Dipropylmethane			
18	Eucalyptone			
19	Daucosterol			

### 6.2 Cardioprotective effects

Aconitum alkaloids (AAs) exhibit cardiovascular protective effects through complementary antioxidant and hemodynamic regulatory mechanisms. In myocardial ischemia–reperfusion injury models, AA enhances myocardial antioxidant capacity by upregulating copper-zinc superoxide dismutase (Cu–ZnSOD) mRNA expression, thereby mitigating oxidative damage (Yin et al., 2005). Beyond antioxidant activity, AAs also demonstrate significant dose-dependent antihypertensive properties. Li and Guo (2019) used Sprague-Dawley rats to systematically evaluate the cardiovascular activity of ethanol-extracted total alkaloids from ATS. A blank control group treated with normal saline combined with heparin was established, along with experimental groups at doses of 150, 200, and 250 mg/kg. The carotid artery intubation method was adopted to continuously monitor blood pressure changes in rats for 30 min. The total alkaloids exerted a dose-dependent hypotensive effect, which could significantly reduce systolic and diastolic blood pressure with mild efficacy without causing drastic hemodynamic fluctuations, showing favorable cardiovascular protective potential. Nevertheless, this study only preliminarily explored the hypotensive effect of total alkaloids without clarifying the specific underlying mechanisms. Only blood pressure was detected, and analyses on heart rate, myocardial function and other related indicators were lacking. In addition, long-term efficacy and safety experiments were not performed, and the effective metabolite components were not screened out, leading to shallow research depth. According to Zhao et al. (2024), structure–activity relationship research on aconitine-type C_19_-diterpenoid alkaloids further interprets the material basis of such cardioprotective effects. The intact C_19_ diterpenoid skeleton together with hydroxyl or methoxy substitutions at C-1, C-3, and C-15α positions contributes to cardiac protective activity, whereas the acetate substituent at C-8 is the critical toxic moiety triggering cardiac arrhythmia. In contrast, lycoctonine-type C_19_-diterpenoid alkaloids have no obvious cardiotonic activity, which provides important structural evidence for subsequent screening of low-toxicity cardioactive metabolites from ATS alkaloids.

Overall, total alkaloids from ATS possess promising cardiovascular protective effects and can regulate blood pressure steadily. However, current studies remain limited by an incomplete understanding of the underlying mechanisms and reliance on single detection indicators; therefore, further in-depth investigations are urgently needed.

### 6.3 Antimicrobial effects

ATS, a traditional heat-clearing and detoxifying agent, exhibits notable antibacterial and antiviral activities as confirmed by modern pharmacological studies. Its extracts show broad-spectrum antibacterial effects against both Gram-positive and Gram-negative bacteria. Zhang et al. (2010) evaluated the *in vitro* antimicrobial activities of aqueous and ethanol extracts of ATS against eight bacterial and fungal strains (including *Staphylococcus aureus*, methicillin-resistant *Staphylococcus epidermidis*, *Escherichia coli*, *Pseudomonas aeruginosa*, and *Candida albicans*) using agar diffusion and broth microdilution methods. Levofloxacin and fluconazole served as positive controls for bacteria and *C. albicans*, respectively, while the corresponding solvents were used as negative controls. Both extracts inhibited a broad spectrum of Gram-positive bacteria, Gram-negative bacteria, and *C. albicans*. Notably, the aqueous extract produced inhibition zones of up to 20 mm against *S. aureus* and *E. coli* and was more active against *P. aeruginosa* and methicillin-resistant *S. epidermidis* than the ethanol extract.

Fu (2008) further assessed the minimum inhibitory concentrations (MICs) of different fractions of ATS via microdilution, including volatile oil, water-soluble alkaloids, fat-soluble alkaloids, and total ethanol extract, each tested at a range of concentrations. The study included blank, experimental, positive (levofloxacin/fluconazole), and negative (sterile water/dimethyl sulfoxide) controls. Volatile oil exhibited the strongest activity, with MICs as low as 0.125–0.25 mg/mL, followed by water-soluble alkaloids, fat-soluble alkaloids, and total ethanol extract.

Although these studies demonstrate the antimicrobial potential of ATS, they have several limitations. Zhang et al. (2010) provided insufficient details on control conditions and did not explore mechanisms or active components beyond inhibition zones and MICs. Fu (2008) compared different fractions but did not include *in vivo* efficacy, safety, or time-kill studies, and neither study identified specific molecular targets, limiting clinical translation. Collectively, these data support ATS traditional applications in botanical drug. Its volatile oils and alkaloids warrant further development as novel anti-infective candidates, particularly for drug-resistant pathogens.

### 6.4 Protective effects against acute lung injury

Modern pharmacological studies further indicate the organ-protective potential of ATS. Wu et al. (2014) investigated the protective effects and mechanisms of AAs, prepared by 95% ethanol reflux extraction and chloroform purification, in a rat model of lipopolysaccharide (LPS)-induced acute lung injury (ALI). Rats were divided into six groups: normal control, AA alone, LPS model, LPS + dexamethasone (positive control), LPS + high-dose AA, and LPS + low-dose AA. AA significantly ameliorated the LPS-induced decrease in arterial partial pressure of oxygen and oxygenation index (PaO_2_/FiO_2_), reduced the lung wet/dry weight ratio, and attenuated pulmonary edema. Meanwhile, AA decreased inflammatory cell infiltration (total cells, neutrophils, and lymphocytes) in bronchoalveolar lavage fluid, lowered myeloperoxidase (MPO) activity in lung tissue, suppressed the release of pro-inflammatory cytokines including TNF-α, IL-6, and IL-1β, and markedly inhibited the activation of nuclear factor-kappa B p65 (NF-κB p65) in the lungs. Histopathological examination further confirmed that AA alleviated LPS-induced inflammatory infiltration, alveolar damage, and edema. These findings indicate that AA exerts a significant protective effect against LPS-induced ALI, which is associated with the inhibition of the NF-κB pathway, reduction of pro-inflammatory cytokine release, and suppression of neutrophil infiltration. Consistent with these results, Pérez et al. (2012) reported that reduced MPO activity correlates with lung injury remission, collectively confirming that alkaloids from ATS protect against LPS-induced ALI through multiple pathways, including NF-κB signaling inhibition, attenuation of inflammatory-cell infiltration, and suppression of pro-inflammatory factor release.

**FIGURE 9:**
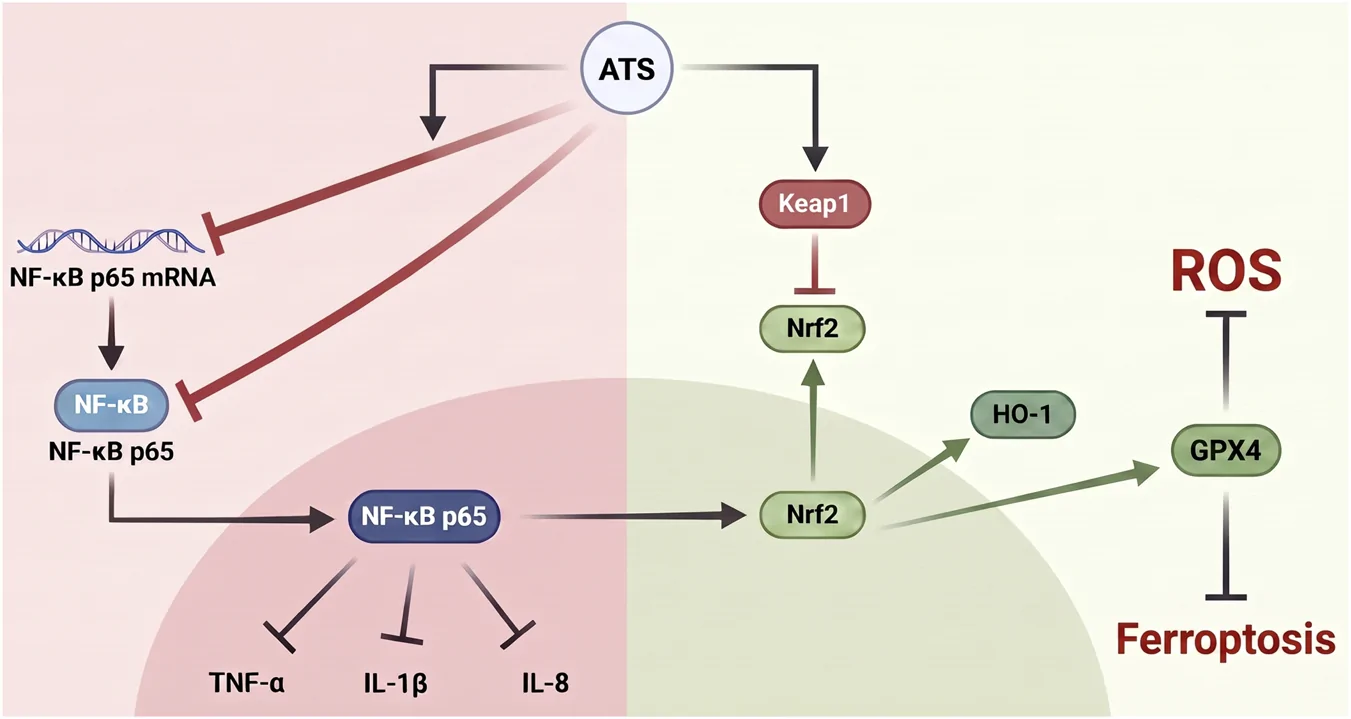
Protective mechanisms of *Aconitum tanguticum* (Maxim.) Stapf. against acute lung injury (Meng et al., 2025). Abbreviations: ATS, *Aconitum tanguticum* (Maxim.) Stapf.; NF-κB, Nuclear factor-kappa B; p65, NF-κB subunit p65; TNF-α, Tumor necrosis factor-alpha; IL-1β, Interleukin-1 beta; IL-8, Interleukin-8; Keap1, Kelch-like ECH-associated protein 1; Nrf2, Nuclear factor erythroid 2-related factor 2; HO-1, Heme oxygenase-1; ROS, Reactive oxygen species; GPX4, Glutathione peroxidase 4.

Building on these findings, Meng et al. (2025) further demonstrated that ATS exerts anti-inflammatory effects by regulating both inflammatory and antioxidant pathways. LPS stimulation activates NF-κB p65 signaling in alveolar epithelial cells and lung tissues, upregulates pro-inflammatory factor expression, and triggers a pulmonary inflammatory storm. Meanwhile, LPS induces excessive accumulation of reactive oxygen species (ROS), which further amplifies inflammatory responses. ATS inhibits the NF-κB pathway to reduce pro-inflammatory factor secretion. It also activates the Kelch-like ECH-associated protein 1/nuclear factor erythroid 2-related factor 2/heme oxygenase-1 (Keap1/Nrf2/HO-1) antioxidant pathway, enhances antioxidant capacity, and eliminates excessive ROS (Figure 9). Collectively, these effects alleviate LPS-induced ALI through synergistic multi-target actions. Published phytochemical research has isolated a series of characteristic C19 norditerpenoid alkaloids from AST, mainly including 6-benzoylheteratisine and atisine. These compounds, characterized by an intact lactone ring and C-14 benzoyl substitution, exhibit potent inhibitory effects on the release of TNF-α and IL-1β (Ye et al., 2023). Existing bioassay data provides preliminary insights into the structure–activity relationship of ATS alkaloids. An intact lactone skeleton has been shown to be essential for anti-inflammatory activity, whereas a benzoyl substituent at C14 markedly enhances potency against inflammatory cascades mediated by NF-κB/Nrf2 pathways, which may contribute to the overall lung-protective effects of ATS total alkaloids.

Collectively, these studies confirm that ATS total alkaloids exert anti-inflammatory protective effects against LPS-induced ALI, providing pharmacological evidence for the traditional efficacy of ATS in clearing heat and detoxifying toxins. They also clarify the dual-pathway anti-inflammatory mechanism of ATS, offering a theoretical foundation for drug development and clinical intervention in ALI and highlighting its promising potential as a candidate drug for the prevention and treatment of pulmonary inflammation. However, current studies have only primarily focused on total alkaloids without identifying the specific active metabolites and have lacked detailed mechanistic investigations, safety evaluations, and multi-model validations. These limitations warrant further investigation in future studies.

### 6.5 Gastroprotective effects

Acute hemorrhagic enteritis (AHE) induces TNF-α and ILs secretion by intestinal mucosal macrophages, inflammation, increased cell membrane permeability and exudation, mucosal ulceration, necrosis, and shedding, leading to impaired intestinal mechanical barrier function. High levels of TNF-α lead to excessive production of nitric oxide, which in turn can cause cell destruction and tissue damage (Song et al., 2009; Wang et al., 2013).

Against this pathological background, Wu (2015) investigated the protective effects of AA in an AHE rat model, establishing five experimental groups: normal control, model control, AA control, positive control (sulfasalazine), and high- and low-dose AA. AA significantly alleviated key AHE symptoms including diarrhea and body weight loss and reduced macroscopic and microscopic injury scores of the ileal mucosa. Mechanistically, AA exerted these effects by inhibiting the release of serum inflammatory factors such as TNF-α and nitric oxide, decreasing the activities of intestinal tissue diamine oxidase and MPO, and downregulating intercellular cell adhesion molecule-1 expression, thereby reducing inflammatory infiltration and pathological damage to the intestinal mucosa. Wu (2015) also confirmed that high-dose aspirin could reduce inflammatory markers and protect intestinal mucosal barriers in a methotrexate-induced rat model, providing experimental evidence for its traditional heat-clearing and detoxifying effects. However, this study only focused on total alkaloids without identifying specific active ingredients, and the regulatory details of the NF-κB inflammatory pathway were not further clarified. Furthermore, the study lacked data on long-term toxicity, pharmacokinetics, and clinical correlations, highlighting key directions for future research.

**FIGURE 10:**
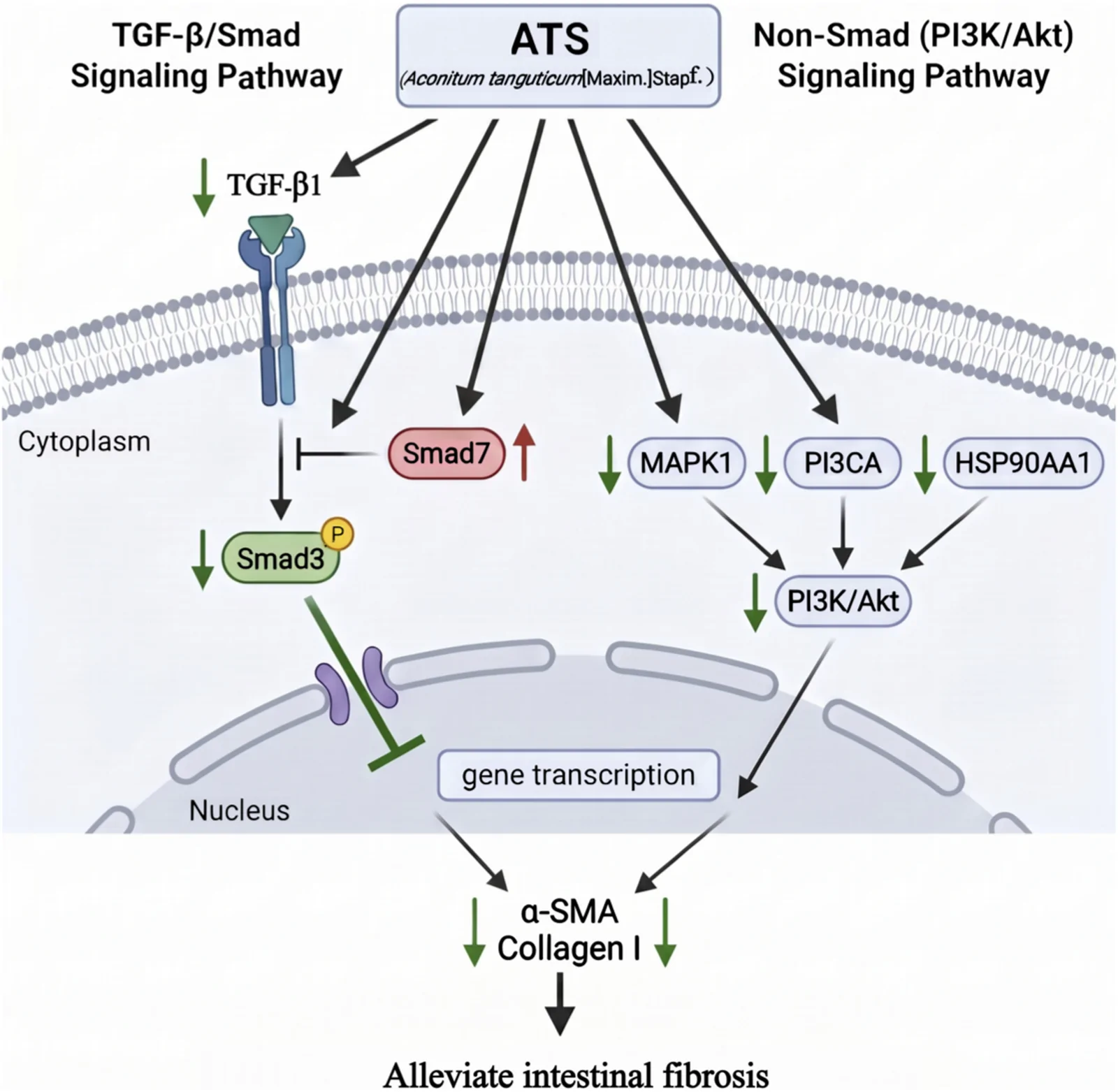
Mechanism of *Aconitum tanguticum* (Maxim.) Stapf. in treating intestinal fibrosis (Cui et al., 2024). Abbreviations: ATS, *Aconitum tanguticum* (Maxim.) Stapf.; TGF-β, Transforming growth factor-beta; TGF-β1, Transforming growth factor-beta 1; Smad, Sma- and Mad-related protein; Smad3, Smad family member 3; Smad7, Smad family member 7; MAPK1, Mitogen-activated protein kinase 1; PI3CA, Phosphatidylinositol-4,5-bisphosphate 3-kinase catalytic subunit alpha; PI3K, Phosphatidylinositol 3-kinase; Akt, Protein kinase B; HSP90AA1, Heat shock protein 90 alpha family class A member 1; α-SMA, Alpha-smooth muscle actin; Collagen I, Type I collagen.

Beyond acute inflammatory injury, chronic intestinal inflammation can also lead to intestinal fibrosis, a condition addressed by recent work. Cui et al. (2024) demonstrated that ATS exerts anti-fibrotic effects by simultaneously regulating the transforming growth factor-β/Smad (TGF-β/Smad) and non-Smad (PI3K/Akt) signaling pathways. In the canonical TGF-β/Smad pathway, ATS downregulates the expression of TGF-β1 and Smad3, upregulates the negative regulator Smad7, and blocks the nuclear translocation of Smad3. In the non-Smad pathway, ATS suppresses excessive activation of the downstream PI3K/Akt pathway by reducing the expression of mitogen-activated protein kinase 1 (MAPK1), phosphatidylinositol-4,5-bisphosphate 3-kinase catalytic subunit alpha (PI3CA), and heat shock protein 90 alpha family class A member 1 (HSP90AA1). The synergistic regulation of these two pathways ultimately reduces the expression of the fibrotic markers alpha-smooth muscle actin (α-SMA) and collagen I, inhibits fibroblast activation and extracellular matrix deposition, and alleviates the progression of intestinal fibrosis. Based on this dual-pathway regulatory mechanism, further studies are warranted to explore the efficacy of ATS at different pathological stages and its dose–response relationships. Such studies would facilitate the translation of these findings from basic research to clinical applications and may help establish novel strategies for the prevention and treatment of intestinal fibrosis (Figure 10).

### 6.6 Neuroprotective effects

Beyond its intestinal protective effects, ATS also exhibits neuroprotective potential. Bai (2022) conducted a series of experiments to explore the neuroprotective function of Bangga. *In vitro* assays showed that the effective concentration range of its active ingredients (total alkaloids and monomeric diterpenoid alkaloids) was 1–100 μmol/L, with 10 μmol/L being the lowest effective concentration. In a scopolamine-induced dementia mouse model, animals were given daily doses of 50–200 mg/kg, and 100 mg/kg per day achieved the best therapeutic effect. To provide a comprehensive investigation, the study combined *in vitro* cell models (BV-2 mouse microglial cells and SH-SY5Y human neuroblastoma cells), *in vivo* cognitive impairment animal models, and network pharmacology prediction. Negative controls and donepezil-treated positive controls were set in all experimental groups. The *in vitro* treatment lasted 24–48 h, and continuous intragastric administration was performed for 14 days *in vivo*. The purity of test samples (total alkaloids ≥85%, monomeric compounds ≥95%) and key pharmacological indexes were fully recorded, covering acetylcholinesterase activity, cell viability, inflammatory factors, and animal behavioral performance. Collectively, all experimental models were designed to target cognitive dysfunction and neuroinflammation.

However, the research findings have mostly been derived from network pharmacology prediction and indirect experiments, and core signaling pathways have not been verified at the *in vivo* protein level. In addition, the structure–activity relationship and target characteristics of monomeric components have not been thoroughly discussed, and systematic long-term toxicity and safety evaluation data are also missing.

Therefore, to facilitate the clinical translation of the neuroprotective effects of ATS, future research should focus on clarifying the molecular targets and mechanisms of action of the core monomeric compounds, evaluating their efficacy in clinically relevant models of Alzheimer’s disease and conducting systematic safety evaluation.

## 7 Toxicity of *Aconitum tanguticum* (Maxim.) Stapf

Aconitum botanical drugs mainly contain diester diterpenoid alkaloids, among which aconitine, mesaconitine, and hypaconitine are the principal toxic constituents. These compounds exert toxic effects by activating sodium channels, disrupting calcium homeostasis, inhibiting Na^+^/K^+^-ATPase activity, triggering oxidative stress, and promoting connexin 43 dephosphorylation, thereby causing injury to the cardiac, nervous, reproductive, digestive, and respiratory systems and exhibiting marked organ-specific toxicity. Clinical poisoning cases have demonstrated the severe safety risks of these alkaloids (Liu et al., 2016). Zhang (2023) retrospectively analyzed 160 patients with acute AA poisoning and found that most cases were caused by ingestion of stewed Aconitum roots or Aconitum-containing medicinal liquor. The main clinical symptoms included numbness, arrhythmia, and gastrointestinal disorders. Among all patients, 97.5% exhibited multiple symptoms, 47.5% had disturbance of consciousness, and 71.25% developed various types of arrhythmia, with an overall mortality rate of 5.63%. These findings indicate that AAs may result in life-threatening multi-system damage.

Traditional Tibetan medicine achieves detoxification while retaining pharmacological activity via heating and auxiliary material processing, which converts highly toxic diester alkaloids into low-toxicity monoester or amine-type alkaloids. Representative processing methods include Terminalia chebula processing, highland barley wine processing, and oil emulsion processing. Medicinal compatibility is the primary detoxification strategy in traditional Chinese medicine (Wang et al., 2017). Peng et al. (2023) adopted metabolomics combined with network pharmacology in healthy rats and heart failure rat models and confirmed the state-dependent toxicity-reducing and efficacy-enhancing effects of the phytopharmaceutical pair of *Aconitum carmichaelii* Debeaux (Ranunculaceae) and *Glycyrrhiza uralensis* Fisch. ex DC. (Fabaceae). Under physiological conditions, this botanical drug pair ameliorates metabolic disorders and reduces toxic reactions. Under pathological conditions, it regulates inflammatory responses and energy metabolism and exerts myocardial protection to enhance therapeutic effects. Metabolomics technology has systematically revealed the chemical and biological basis underlying the “efficacy–toxicity” transformation of AAs (Peng et al., 2023; Ji et al., 2023; Li et al., 2023) and elucidated the mechanisms of processing-induced detoxification and compatibility-mediated synergism at the metabolic pathway level (Sun et al., 2026), providing scientific evidence for the safe clinical use of these drugs.

ATS does not contain highly toxic diester alkaloids and is characterized by low toxicity and a favorable safety profile. The daily dosage of 2–4 g recommended in Tibetan medicine is considered safe for clinical use. Among commonly used *Aconitum* medicinal materials, the toxicity is as follows: *Aconitum brachypodum* Diels (Ranunculaceae) > *Aconitum kusnezoffii* Rchb. (Ranunculaceae) > *Aconitum carmichaelii* Debeaux (Ranunculaceae) > *Aconitum tanguticum* (Maxim.) Stapf (Ranunculaceae) (Liu et al., 2016). Ren-Qing-Zhang-Jiao, a Tibetan patent medicine containing ATS, was administered to 42 patients with chronic idiopathic diarrhea and abdominal pain. Satisfactory therapeutic effects were observed in 35 cases. This drug also relieved accompanying symptoms such as hypoalbuminemia and insomnia without evident adverse reactions, confirming its safety and efficacy (Wuse, 2010).

An-Er-Ning-Ke-Li consists of Bambusae Concertio Silicea (Poaceae), *Carthamus tinctorius* L. (Asteraceae), Bovis Calculus (*Bos taurus domesticus* Gmelin), and other medicinal ingredients. In traditional medicine, it is used to clear heat, dispel wind, resolve phlegm, and relieve cough. Modern pharmacological studies have demonstrated its favorable analgesic, anti-inflammatory, bacteriostatic, and anti-asthmatic activities (Shi et al., 2020). Wei et al. (2022) conducted a randomized, double-blind, single-dummy, placebo-controlled multicenter clinical trial to evaluate the efficacy of An-Er-Ning-Ke-Li in reducing antibiotic use in children with community-acquired pneumonia. The granules significantly shortened the duration of fever and cough, increased the clinical cure rate, and reduced the course of antibiotic treatment with adequate safety. This study provided evidence for integrated Chinese and Western medicine therapy and rational antibiotic use in pediatric pneumonia. Notably, this research focused on the adjuvant application of proprietary Chinese medicines, which differs from the direct clinical use of Aconitum botanical drugs. At present, human clinical trials on Aconitum botanical drugs remain limited; therefore, their clinical value should not be overstated. In addition, regulatory restrictions, inherent toxicity risks, and the lack of standardized processing techniques hinder their clinical promotion.

The severe cardiotoxicity, neurotoxicity, and hepatotoxicity associated with diterpenoid AAs limit their clinical application. Future research should explore their structural characteristics, synthetic biology, and toxicological mechanisms. Combined with structural modification and targeted delivery technology, pharmacokinetic properties can be optimized, effective strategies for detoxification can be established, and efficacy can be improved. More standardized clinical trials are needed to define the safe dosage range. Such studies would provide a foundation for the further development and clinical application of Aconitum-derived medicines (Feng et al., 2026).

## 8 Summary and future prospects

In this study, we systematically reviewed the traditional uses, botanical characteristics, chemical constituents, and pharmacological effects of ATS, which has been commonly used in Tibetan medicine preparations for thousands of years and is deeply appreciated by all ethnic groups (Bai et al., 2023).

ATS is included in the Zhong-Hua-Ren-Min-Gong-He-Guo-Wei-Sheng-Bu-Yao-Pin-Biao-Zhun. Studies on its traditional uses and clinical applications report effects such as clearing heat and detoxification, eliminating old heat, and treating hepatobiliary fever, influenza, plague, hepatitis, cholecystitis, blood disease, and yellow water disease. At the beginning of the severe acute respiratory syndrome (SARS) epidemic, Tibet, Qinghai, and other provinces that use Tibetan medicines recommended formulations containing ATS (Shi-Er-Wei-Yi-Shou-San, Er-Shi-Wu-Wei-Fei-Bing-Jiao-Nang, Liu-Gan-Wan, and Shi-Er-Wei-Zhu-Yao-San) for infection prevention and control programs. Shi-Er-Wei-Yi-Shou-San was widely used during the SARS outbreak and reportedly demonstrated good preventive and therapeutic effects against the inflammatory storm caused by viral infection (Downs, 2021).

However, Tibetan medicines are not widely used in China for the following reasons. First, most clinical studies on efficacy and safety are non-randomized comparative trials (before–after) with clear limitations, including a lack of blank controls and multicenter randomized controls. Second, preparations are simple, often oral or external decoctions, pills, and powders, with unpleasant odors, high dosages, and large pills that are difficult to swallow, especially for children and older adults. Third, research on toxicity is limited; for example, as noted above, an effective antidote for aconite poisoning is lacking in clinical practice. In addition, components of Tibetan medicine preparations are complex and variable, and the same prescription may appear in different formulations across texts, thereby complicating the establishment of unified standards (Fan et al., 2023; Li et al., 2024). These issues should be addressed as follows: first, experts and scholars should develop new research standards for Tibetan medicinal preparations, using modern detection technologies to establish PCR-fingerprinting (Fan et al., 2023) and conduct in-depth studies; second, dosages should be reformulated as sustained-release oral preparations to improve bioavailability and patient compliance; and third, toxicological research should be strengthened to guide rational clinical use and avoid or reduce adverse reactions.

Overall, as a core ethnic Tibetan medicine, ATS has gained increased recognition for its anti-inflammatory, antiviral, and other pharmacological activities, as well as its clinical applications. However, it still faces significant limitations. Differences in total alkaloid extraction methods across studies may lead to fluctuations in composition and activity assessments. This can affect the comparability of results. Research on chemical composition focuses on crude extracts of alkaloids, whereas studies on specific alkaloids and other monomeric components remain limited. To address these challenges and facilitate translational progress, future research should prioritize robust chemical standardization and quality control based on metabolomic profiling and fingerprinting approaches. Key priorities include: (1) implementing high-performance liquid chromatography/liquid chromatography–mass spectrometry-based quality control workflows to quantify marker compounds (e.g., aconitine-type alkaloids) across different batches, thereby minimizing batch-to-batch variability and ensuring material consistency (Kellogg et al., 2025); (2) developing authentication strategies, including DNA barcoding and multi-component fingerprinting, to verify the botanical identity of ATS and prevent adulteration (Dev et al., 2021); and (3) aligning these analytical frameworks with Good Manufacturing Practice principles to support the scalable and reproducible production of standardized extracts. This critical refocusing is essential for transforming traditional knowledge into evidence-based application.

Research on the pharmacological mechanisms of ATS remains at the preliminary level, characterized by apparent mechanisms, but lacks in-depth exploration of molecular mechanisms. Future research breakthroughs are needed across multiple dimensions. Beyond deepening investigations of active components and mechanisms of action and refining toxicity evaluation systems, several advanced approaches may accelerate translational development. For example, nanotechnology-based drug delivery systems (e.g., nanocarriers) may improve the solubility, targeted delivery, and safety profile of ATS alkaloids, thereby addressing key limitations of conventional formulations (Fattahi et al., 2020). Computational drug discovery and AI-assisted screening may facilitate the rapid identification and prioritization of bioactive monomeric compounds, supporting targeted component isolation and structure–activity relationship studies. Pharmacokinetic modeling may be used to characterize the absorption, distribution, metabolism, and excretion of key constituents, thereby informing rational dosing regimens and reducing toxicity risks. Multi-omics approaches (including transcriptomics, proteomics, and metabolomics) may further elucidate the complex molecular mechanisms underlying the anti-inflammatory and anti-viral activities of ATS (Fan et al., 2020). Collectively, the integration of these advances into a streamlined translational drug development pipeline may help bridge the gap between preclinical and clinical research, facilitating the transition of ATS from traditional use to evidence-based clinical application. By summarizing the traditional uses, botanical characteristics, chemical composition, and pharmacological effects of ATS, this review highlights its importance in traditional prescriptions and its potential for modern clinical application. The findings underscore the importance of conserving traditional medicinal resources and provide a basis for the rational use and future development of ATS.
